# Supplementary material for: Phasing Diploid Genome Assembly Graphs with Single-Cell Strand Sequencing
Source: bioRxiv. 2024 Jun 20:2024.02.15.580432. Originally published 2024 Feb 16. Preprint. [Version 2] doi: 10.1101/2024.02.15.580432 (PMC10962706; doi:10.1101/2024.02.15.580432)
Supplement: Supplement 1 [file media-1.pdf]

Supplement

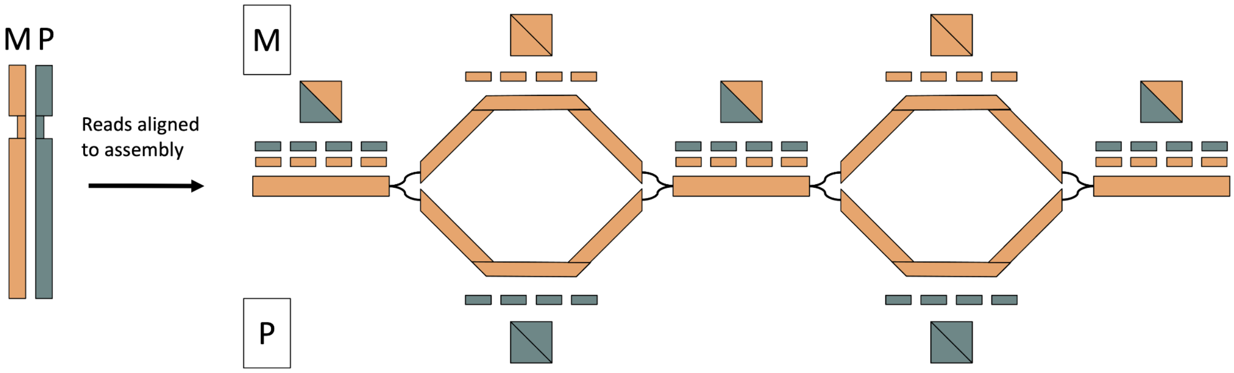

Figure S1. Strand-seq Phasing Signal. A reads from a WC Strand-seq library (left) are aligned to the unitigs of a chromosome (right). Orange shapes correspond to Watson orientation, and green shapes correspond to Crick orientation. The graph unitigs and maternal reads are all in Watson orientation, while the paternal reads are all in Crick orientation. Consequently, sequence unique to the maternal haplotype will receive only "forward" alignments (orange box), while sequence unique to the paternal haplotype will receive only "reverse" alignments (green box). Sequence shared between the haplotypes receives a 50/50 mix of forward and reverse alignments (green\orange box)

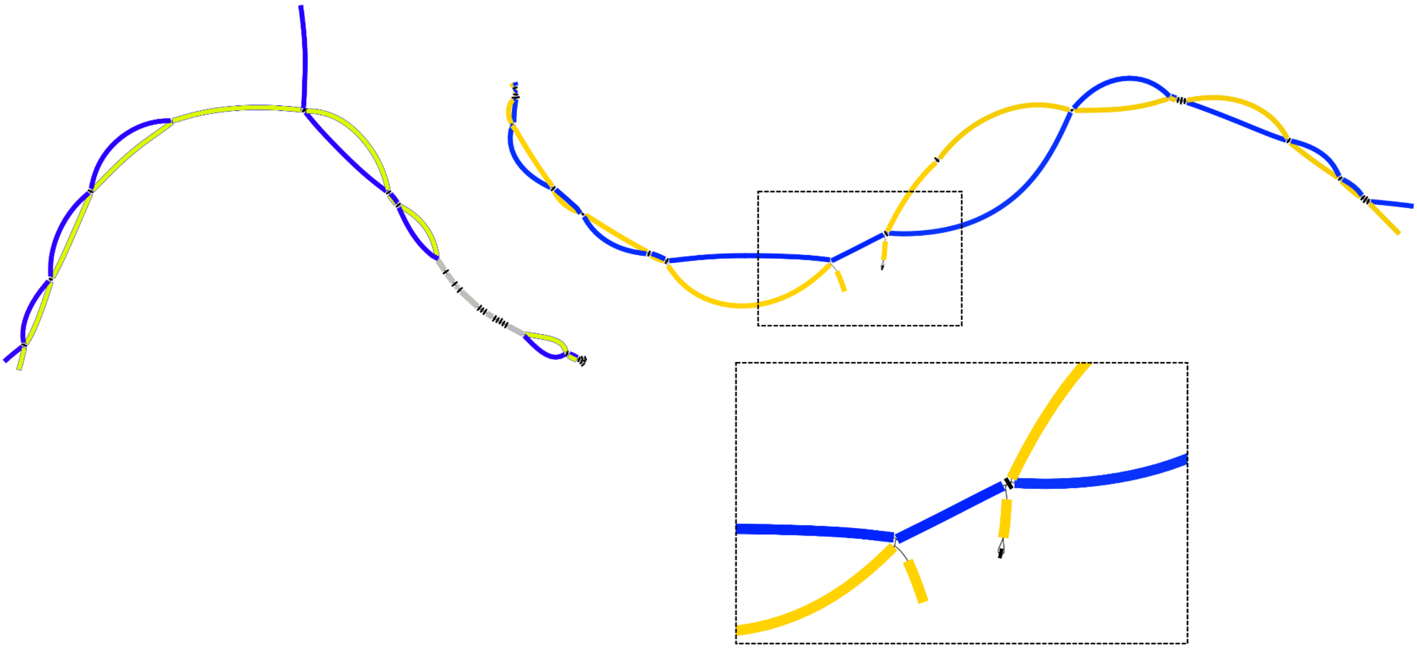

Figure S2. Hairpin-Capped Broken Bubbles interrupt Rukki Scaffolding. Bandage [67] graphs of chromosomes 5 (left) and 1 (right, top) from the Verkko HG00733 assembly both show broken bubbles. On each connected component, the blue or yellow shade corresponds to a haplotype as inferred by Graphasing, grey corresponds to homozygous nodes, and black to nodes without inferred phasing signal. The blue haplotype on chromosome 5 is scaffolded in one path, but the yellow haplotype of chromosome 1 is represented with two scaffolds, one on

either side of the broken bubble, and is the only pictured haplotype not connected in a single path. Zooming in (right, bottom) shows that one end of the broken bubble is "capped" by a single small node in a hairpin structure.

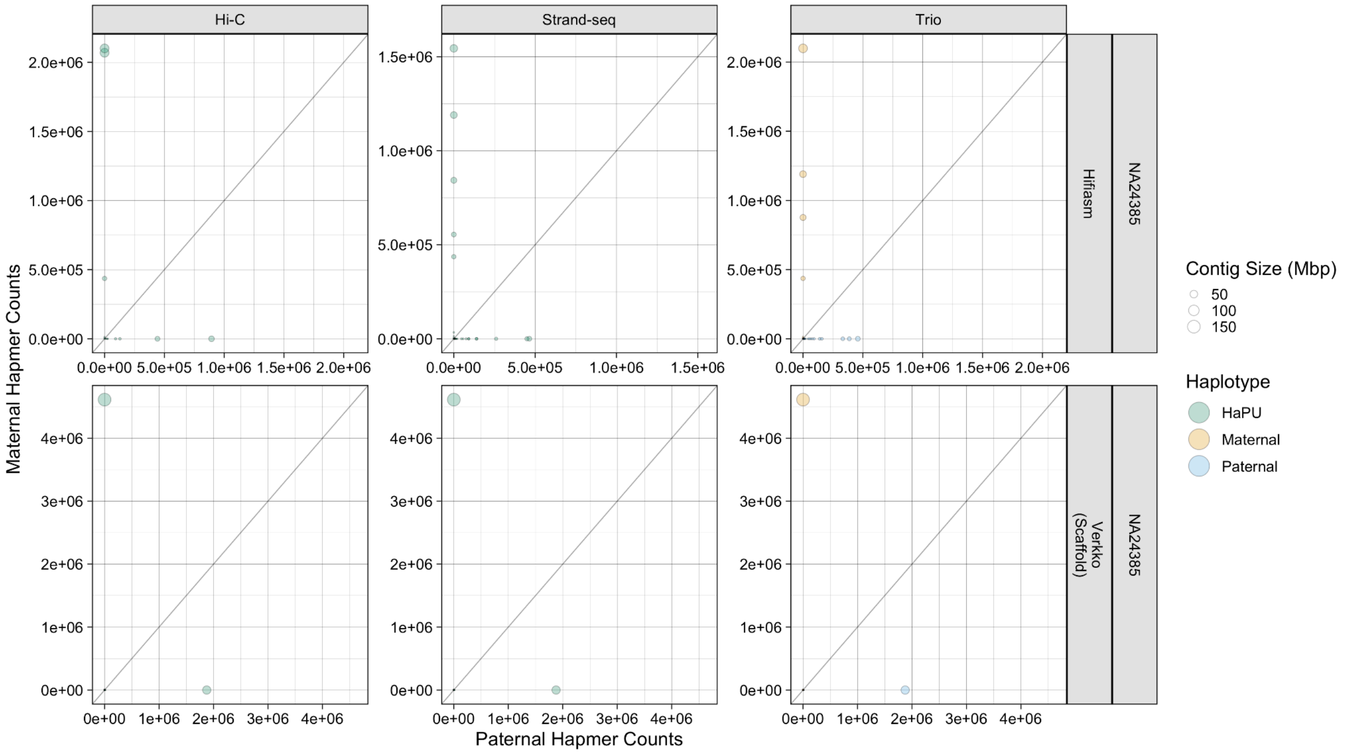

Figure S3. Sex Chromosome Hap-mer Blob Plots. NA24385 contigs aligning to the X and Y chromosomes are plotted.

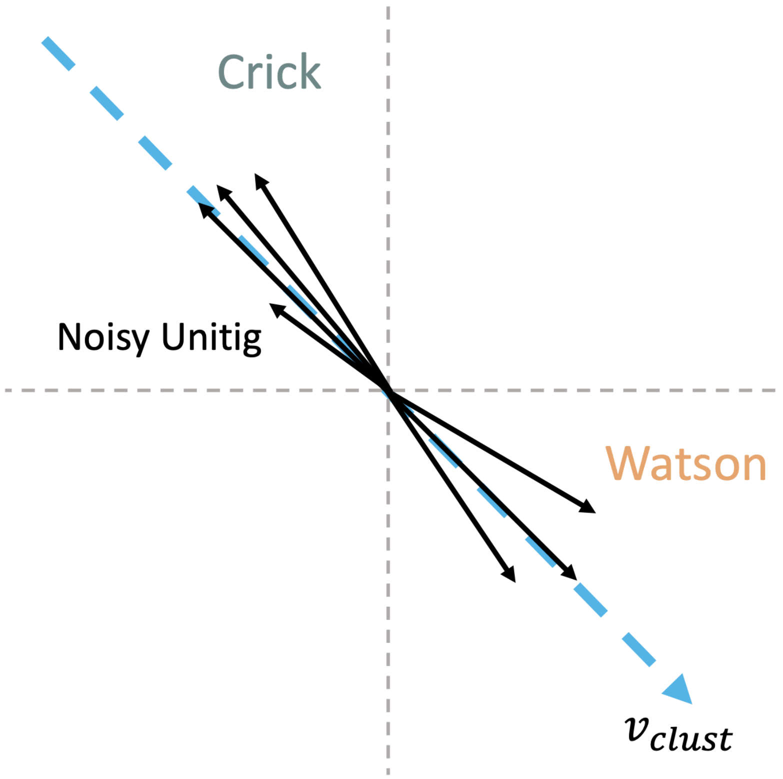

Figure S4. Behavior of Absolute SSF Calculated with bwa mem alignments. Each axis corresponds to a different Strand-seq library. Unitigs from the same chromosome, but in opposite orientation will point in opposite directions along the same cluster vector (blue dashed vector), but are nonetheless clustered together when the absolute cosine similarity is used. A unitig that contains noisy, degenerate sequence is skewed and shrunk towards the 0 vector, but still points along its corresponding cluster vector.

	Hap 1	Hap 2
WC Library 1	Watson	Crick
WC Library 2	Crick	Watson
WC Library 3	Crick	Watson
WC Library 4	Watson	Crick

➡

	Hap 1	Hap 2
WC Library 1	Crick	Watson
WC Library 2	Crick	Watson
WC Library 3	Crick	Watson
WC Library 4	Crick	Watson

Figure S5. Read Labeling for Proper Strand-seq Library Pooling. In each Strand-seq library, the labels (Watson, Crick) of the reads aligning to each haplotype depend on the orientation of the unitigs and the inherited Strand-state of the unmatched strand state library (WC vs. CW library). However, swapping the labels of reads does not affect the steps before library pooling, and so the labeling within each library can safely be treated as if it were arbitrary. Left: Each haplotype receives only Watson or only Crick alignments within each library. However, the pooled library assigns each haplotype a 50/50 ratio of Watson and Crick alignments and fails to reflect the behavior at the individual library level. Right: The labels of libraries 1 and 4 are swapped, and the pooled library now also assigns reads of only one label to each haplotype.

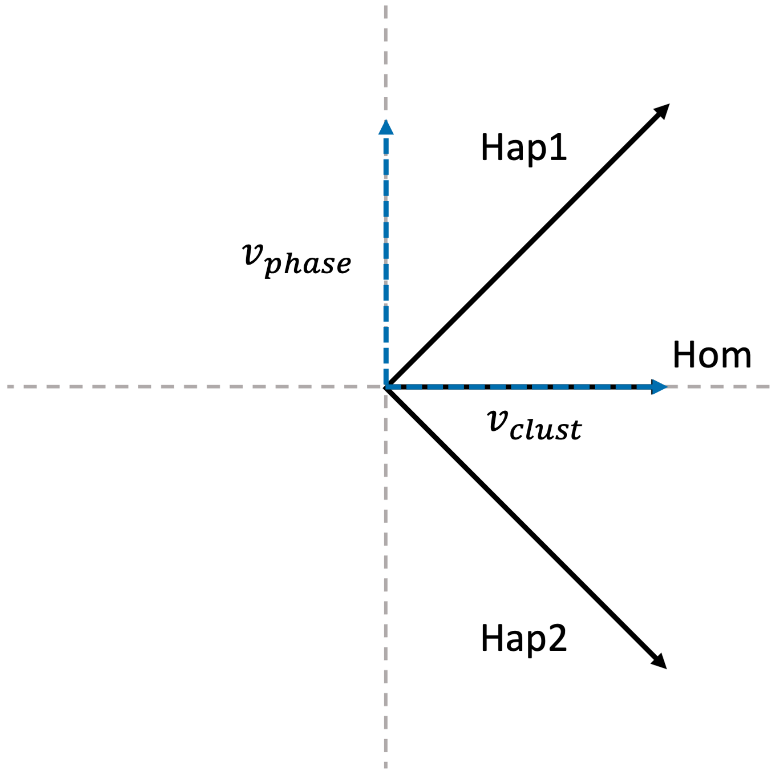

Figure S6. Each axis corresponds to a different Strand-seq library. When the SSF is calculated with phase-informative reads, the unitig vectors (black arrows) separate from the cluster vector according to the haplotype. The difference between the haplotype 1 and haplotype 2 vectors defines the phase vector, which is used to pool Strand-seq libraries. The vectors of unitigs that contain homozygous sequence remain parallel to the cluster vector regardless of the reads used to calculate the SSF.

Table S1: Unitig Filtering Statistics

Sample	Assembler	Total Unitigs (n)	Unitigs < 50kbp (n)	Total Assembly Size < 50kbp (%)
NA24385	Verkko hybrid	8,627	4,966	2.2
NA24385	hifiasm hybrid	12,520	4,355	2.5
HG00733	Verkko hybrid	1,882	1,353	0.5
HG00733	hifiasm hybrid	4,579	2,026	1.2

Table S2: NA24385 Strand-seq library quality annotations:

File 1	File 2	Annotation
HWVTJAFXY_HG002x1xHG002x2_19s005136-1-1_Hasenfeld_lane1HG002x01PE20301_1_sequence.txt.gz	HWVTJAFXY_HG002x1xHG002x2_19s005136-1-1_Hasenfeld_lane1HG002x01PE20301_2_sequence.txt.gz	HighQuality
HWVTJAFXY_HG002x1xHG002x2_19s005136-1-1_Hasenfeld_lane1HG002x01PE20303_1_sequence.txt.gz	HWVTJAFXY_HG002x1xHG002x2_19s005136-1-1_Hasenfeld_lane1HG002x01PE20303_2_sequence.txt.gz	HighQuality

HWVTJAFXY_HG002x1xHG002x2_19s005136-1-1_Hasenfeld_lane1HG002x01PE20305_1_sequence.txt.gz	HWVTJAFXY_HG002x1xHG002x2_19s005136-1-1_Hasenfeld_lane1HG002x01PE20305_2_sequence.txt.gz	HighQuality
HWVTJAFXY_HG002x1xHG002x2_19s005136-1-1_Hasenfeld_lane1HG002x01PE20306_1_sequence.txt.gz	HWVTJAFXY_HG002x1xHG002x2_19s005136-1-1_Hasenfeld_lane1HG002x01PE20306_2_sequence.txt.gz	HighQuality
HWVTJAFXY_HG002x1xHG002x2_19s005136-1-1_Hasenfeld_lane1HG002x01PE20307_1_sequence.txt.gz	HWVTJAFXY_HG002x1xHG002x2_19s005136-1-1_Hasenfeld_lane1HG002x01PE20307_2_sequence.txt.gz	HighQuality
HWVTJAFXY_HG002x1xHG002x2_19s005136-1-1_Hasenfeld_lane1HG002x01PE20308_1_sequence.txt.gz	HWVTJAFXY_HG002x1xHG002x2_19s005136-1-1_Hasenfeld_lane1HG002x01PE20308_2_sequence.txt.gz	HighQuality
HWVTJAFXY_HG002x1xHG002x2_19s005136-1-1_Hasenfeld_lane1HG002x01PE20313_1_sequence.txt.gz	HWVTJAFXY_HG002x1xHG002x2_19s005136-1-1_Hasenfeld_lane1HG002x01PE20313_2_sequence.txt.gz	HighQuality
HWVTJAFXY_HG002x1xHG002x2_19s005136-1-1_Hasenfeld_lane1HG002x01PE20315_1_sequence.txt.gz	HWVTJAFXY_HG002x1xHG002x2_19s005136-1-1_Hasenfeld_lane1HG002x01PE20315_2_sequence.txt.gz	HighQuality
HWVTJAFXY_HG002x1xHG002x2_19s005136-1-1_Hasenfeld_lane1HG002x01PE20318_1_sequence.txt.gz	HWVTJAFXY_HG002x1xHG002x2_19s005136-1-1_Hasenfeld_lane1HG002x01PE20318_2_sequence.txt.gz	HighQuality
HWVTJAFXY_HG002x1xHG002x2_19s005136-1-1_Hasenfeld_lane1HG002x01PE20319_1_sequence.txt.gz	HWVTJAFXY_HG002x1xHG002x2_19s005136-1-1_Hasenfeld_lane1HG002x01PE20319_2_sequence.txt.gz	HighQuality
HWVTJAFXY_HG002x1xHG002x2_19s005136-1-1_Hasenfeld_lane1HG002x01PE20325_1_sequence.txt.gz	HWVTJAFXY_HG002x1xHG002x2_19s005136-1-1_Hasenfeld_lane1HG002x01PE20325_2_sequence.txt.gz	HighQuality
HWVTJAFXY_HG002x1xHG002x2_19s005136-1-1_Hasenfeld_lane1HG002x01PE20327_1_sequence.txt.gz	HWVTJAFXY_HG002x1xHG002x2_19s005136-1-1_Hasenfeld_lane1HG002x01PE20327_2_sequence.txt.gz	HighQuality
HWVTJAFXY_HG002x1xHG002x2_19s005136-1-1_Hasenfeld_lane1HG002x01PE20328_1_sequence.txt.gz	HWVTJAFXY_HG002x1xHG002x2_19s005136-1-1_Hasenfeld_lane1HG002x01PE20328_2_sequence.txt.gz	HighQuality
HWVTJAFXY_HG002x1xHG002x2_19s005136-1-1_Hasenfeld_lane1HG002x01PE20329_1_sequence.txt.gz	HWVTJAFXY_HG002x1xHG002x2_19s005136-1-1_Hasenfeld_lane1HG002x01PE20329_2_sequence.txt.gz	HighQuality
HWVTJAFXY_HG002x1xHG002x2_19s005136-1-1_Hasenfeld_lane1HG002x01PE20331_1_sequence.txt.gz	HWVTJAFXY_HG002x1xHG002x2_19s005136-1-1_Hasenfeld_lane1HG002x01PE20331_2_sequence.txt.gz	HighQuality
HWVTJAFXY_HG002x1xHG002x2_19s005136-1-1_Hasenfeld_lane1HG002x01PE20332_1_sequence.txt.gz	HWVTJAFXY_HG002x1xHG002x2_19s005136-1-1_Hasenfeld_lane1HG002x01PE20332_2_sequence.txt.gz	HighQuality
HWVTJAFXY_HG002x1xHG002x2_19s005136-1-1_Hasenfeld_lane1HG002x01PE20334_1_sequence.txt.gz	HWVTJAFXY_HG002x1xHG002x2_19s005136-1-1_Hasenfeld_lane1HG002x01PE20334_2_sequence.txt.gz	HighQuality
HWVTJAFXY_HG002x1xHG002x2_19s005136-1-1_Hasenfeld_lane1HG002x01PE20335_1_sequence.txt.gz	HWVTJAFXY_HG002x1xHG002x2_19s005136-1-1_Hasenfeld_lane1HG002x01PE20335_2_sequence.txt.gz	HighQuality
HWVTJAFXY_HG002x1xHG002x2_19s005136-1-1_Hasenfeld_lane1HG002x01PE20336_1_sequence.txt.gz	HWVTJAFXY_HG002x1xHG002x2_19s005136-1-1_Hasenfeld_lane1HG002x01PE20336_2_sequence.txt.gz	HighQuality
HWVTJAFXY_HG002x1xHG002x2_19s005136-1-1_Hasenfeld_lane1HG002x01PE20337_1_sequence.txt.gz	HWVTJAFXY_HG002x1xHG002x2_19s005136-1-1_Hasenfeld_lane1HG002x01PE20337_2_sequence.txt.gz	HighQuality
HWVTJAFXY_HG002x1xHG002x2_19s005136-1-1_Hasenfeld_lane1HG002x01PE20339_1_sequence.txt.gz	HWVTJAFXY_HG002x1xHG002x2_19s005136-1-1_Hasenfeld_lane1HG002x01PE20339_2_sequence.txt.gz	HighQuality
HWVTJAFXY_HG002x1xHG002x2_19s005136-1-1_Hasenfeld_lane1HG002x01PE20340_1_sequence.txt.gz	HWVTJAFXY_HG002x1xHG002x2_19s005136-1-1_Hasenfeld_lane1HG002x01PE20340_2_sequence.txt.gz	HighQuality
HWVTJAFXY_HG002x1xHG002x2_19s005136-1-1_Hasenfeld_lane1HG002x01PE20341_1_sequence.txt.gz	HWVTJAFXY_HG002x1xHG002x2_19s005136-1-1_Hasenfeld_lane1HG002x01PE20341_2_sequence.txt.gz	HighQuality
HWVTJAFXY_HG002x1xHG002x2_19s005136-1-1_Hasenfeld_lane1HG002x01PE20342_1_sequence.txt.gz	HWVTJAFXY_HG002x1xHG002x2_19s005136-1-1_Hasenfeld_lane1HG002x01PE20342_2_sequence.txt.gz	HighQuality
HWVTJAFXY_HG002x1xHG002x2_19s005136-1-1_Hasenfeld_lane1HG002x01PE20343_1_sequence.txt.gz	HWVTJAFXY_HG002x1xHG002x2_19s005136-1-1_Hasenfeld_lane1HG002x01PE20343_2_sequence.txt.gz	HighQuality
HWVTJAFXY_HG002x1xHG002x2_19s005136-1-1_Hasenfeld_lane1HG002x01PE20345_1_sequence.txt.gz	HWVTJAFXY_HG002x1xHG002x2_19s005136-1-1_Hasenfeld_lane1HG002x01PE20345_2_sequence.txt.gz	HighQuality
HWVTJAFXY_HG002x1xHG002x2_19s005136-1-1_Hasenfeld_lane1HG002x01PE20347_1_sequence.txt.gz	HWVTJAFXY_HG002x1xHG002x2_19s005136-1-1_Hasenfeld_lane1HG002x01PE20347_2_sequence.txt.gz	HighQuality

HWVTJAFXY_HG002x1xHG002x2_19s005136-1-1_Hasenfeld_lane1HG002x01PE20350_1_sequence.txt.gz	HWVTJAFXY_HG002x1xHG002x2_19s005136-1-1_Hasenfeld_lane1HG002x01PE20350_2_sequence.txt.gz	HighQuality
HWVTJAFXY_HG002x1xHG002x2_19s005136-1-1_Hasenfeld_lane1HG002x01PE20351_1_sequence.txt.gz	HWVTJAFXY_HG002x1xHG002x2_19s005136-1-1_Hasenfeld_lane1HG002x01PE20351_2_sequence.txt.gz	HighQuality
HWVTJAFXY_HG002x1xHG002x2_19s005136-1-1_Hasenfeld_lane1HG002x01PE20352_1_sequence.txt.gz	HWVTJAFXY_HG002x1xHG002x2_19s005136-1-1_Hasenfeld_lane1HG002x01PE20352_2_sequence.txt.gz	HighQuality
HWVTJAFXY_HG002x1xHG002x2_19s005136-1-1_Hasenfeld_lane1HG002x01PE20353_1_sequence.txt.gz	HWVTJAFXY_HG002x1xHG002x2_19s005136-1-1_Hasenfeld_lane1HG002x01PE20353_2_sequence.txt.gz	HighQuality
HWVTJAFXY_HG002x1xHG002x2_19s005136-1-1_Hasenfeld_lane1HG002x01PE20355_1_sequence.txt.gz	HWVTJAFXY_HG002x1xHG002x2_19s005136-1-1_Hasenfeld_lane1HG002x01PE20355_2_sequence.txt.gz	HighQuality
HWVTJAFXY_HG002x1xHG002x2_19s005136-1-1_Hasenfeld_lane1HG002x01PE20356_1_sequence.txt.gz	HWVTJAFXY_HG002x1xHG002x2_19s005136-1-1_Hasenfeld_lane1HG002x01PE20356_2_sequence.txt.gz	HighQuality
HWVTJAFXY_HG002x1xHG002x2_19s005136-1-1_Hasenfeld_lane1HG002x01PE20357_1_sequence.txt.gz	HWVTJAFXY_HG002x1xHG002x2_19s005136-1-1_Hasenfeld_lane1HG002x01PE20357_2_sequence.txt.gz	HighQuality
HWVTJAFXY_HG002x1xHG002x2_19s005136-1-1_Hasenfeld_lane1HG002x01PE20358_1_sequence.txt.gz	HWVTJAFXY_HG002x1xHG002x2_19s005136-1-1_Hasenfeld_lane1HG002x01PE20358_2_sequence.txt.gz	HighQuality
HWVTJAFXY_HG002x1xHG002x2_19s005136-1-1_Hasenfeld_lane1HG002x01PE20359_1_sequence.txt.gz	HWVTJAFXY_HG002x1xHG002x2_19s005136-1-1_Hasenfeld_lane1HG002x01PE20359_2_sequence.txt.gz	HighQuality
HWVTJAFXY_HG002x1xHG002x2_19s005136-1-1_Hasenfeld_lane1HG002x01PE20361_1_sequence.txt.gz	HWVTJAFXY_HG002x1xHG002x2_19s005136-1-1_Hasenfeld_lane1HG002x01PE20361_2_sequence.txt.gz	HighQuality
HWVTJAFXY_HG002x1xHG002x2_19s005136-1-1_Hasenfeld_lane1HG002x01PE20362_1_sequence.txt.gz	HWVTJAFXY_HG002x1xHG002x2_19s005136-1-1_Hasenfeld_lane1HG002x01PE20362_2_sequence.txt.gz	HighQuality
HWVTJAFXY_HG002x1xHG002x2_19s005136-1-1_Hasenfeld_lane1HG002x01PE20363_1_sequence.txt.gz	HWVTJAFXY_HG002x1xHG002x2_19s005136-1-1_Hasenfeld_lane1HG002x01PE20363_2_sequence.txt.gz	HighQuality
HWVTJAFXY_HG002x1xHG002x2_19s005136-1-1_Hasenfeld_lane1HG002x01PE20364_1_sequence.txt.gz	HWVTJAFXY_HG002x1xHG002x2_19s005136-1-1_Hasenfeld_lane1HG002x01PE20364_2_sequence.txt.gz	HighQuality
HWVTJAFXY_HG002x1xHG002x2_19s005136-1-1_Hasenfeld_lane1HG002x01PE20367_1_sequence.txt.gz	HWVTJAFXY_HG002x1xHG002x2_19s005136-1-1_Hasenfeld_lane1HG002x01PE20367_2_sequence.txt.gz	HighQuality
HWVTJAFXY_HG002x1xHG002x2_19s005136-1-1_Hasenfeld_lane1HG002x01PE20368_1_sequence.txt.gz	HWVTJAFXY_HG002x1xHG002x2_19s005136-1-1_Hasenfeld_lane1HG002x01PE20368_2_sequence.txt.gz	HighQuality
HWVTJAFXY_HG002x1xHG002x2_19s005136-1-1_Hasenfeld_lane1HG002x01PE20374_1_sequence.txt.gz	HWVTJAFXY_HG002x1xHG002x2_19s005136-1-1_Hasenfeld_lane1HG002x01PE20374_2_sequence.txt.gz	HighQuality
HWVTJAFXY_HG002x1xHG002x2_19s005136-1-1_Hasenfeld_lane1HG002x01PE20376_1_sequence.txt.gz	HWVTJAFXY_HG002x1xHG002x2_19s005136-1-1_Hasenfeld_lane1HG002x01PE20376_2_sequence.txt.gz	HighQuality
HWVTJAFXY_HG002x1xHG002x2_19s005136-1-1_Hasenfeld_lane1HG002x01PE20377_1_sequence.txt.gz	HWVTJAFXY_HG002x1xHG002x2_19s005136-1-1_Hasenfeld_lane1HG002x01PE20377_2_sequence.txt.gz	HighQuality
HWVTJAFXY_HG002x1xHG002x2_19s005136-1-1_Hasenfeld_lane1HG002x01PE20378_1_sequence.txt.gz	HWVTJAFXY_HG002x1xHG002x2_19s005136-1-1_Hasenfeld_lane1HG002x01PE20378_2_sequence.txt.gz	HighQuality
HWVTJAFXY_HG002x1xHG002x2_19s005136-1-1_Hasenfeld_lane1HG002x01PE20379_1_sequence.txt.gz	HWVTJAFXY_HG002x1xHG002x2_19s005136-1-1_Hasenfeld_lane1HG002x01PE20379_2_sequence.txt.gz	HighQuality
HWVTJAFXY_HG002x1xHG002x2_19s005136-1-1_Hasenfeld_lane1HG002x01PE20381_1_sequence.txt.gz	HWVTJAFXY_HG002x1xHG002x2_19s005136-1-1_Hasenfeld_lane1HG002x01PE20381_2_sequence.txt.gz	HighQuality
HWVTJAFXY_HG002x1xHG002x2_19s005136-1-1_Hasenfeld_lane1HG002x01PE20387_1_sequence.txt.gz	HWVTJAFXY_HG002x1xHG002x2_19s005136-1-1_Hasenfeld_lane1HG002x01PE20387_2_sequence.txt.gz	HighQuality
HWVTJAFXY_HG002x1xHG002x2_19s005136-1-1_Hasenfeld_lane1HG002x01PE20388_1_sequence.txt.gz	HWVTJAFXY_HG002x1xHG002x2_19s005136-1-1_Hasenfeld_lane1HG002x01PE20388_2_sequence.txt.gz	HighQuality
HWVTJAFXY_HG002x1xHG002x2_19s005136-1-1_Hasenfeld_lane1HG002x01PE20389_1_sequence.txt.gz	HWVTJAFXY_HG002x1xHG002x2_19s005136-1-1_Hasenfeld_lane1HG002x01PE20389_2_sequence.txt.gz	HighQuality
HWVTJAFXY_HG002x1xHG002x2_19s005136-1-1_Hasenfeld_lane1HG002x01PE20391_1_sequence.txt.gz	HWVTJAFXY_HG002x1xHG002x2_19s005136-1-1_Hasenfeld_lane1HG002x01PE20391_2_sequence.txt.gz	HighQuality

HWVTJAFXY_HG002x1xHG002x2_19s005136-1-1_Hasenfeld_lane1HG002x01PE20392_1_sequence.txt.gz	HWVTJAFXY_HG002x1xHG002x2_19s005136-1-1_Hasenfeld_lane1HG002x01PE20392_2_sequence.txt.gz	HighQuality
HWVTJAFXY_HG002x1xHG002x2_19s005136-1-1_Hasenfeld_lane1HG002x01PE20393_1_sequence.txt.gz	HWVTJAFXY_HG002x1xHG002x2_19s005136-1-1_Hasenfeld_lane1HG002x01PE20393_2_sequence.txt.gz	HighQuality
HWVTJAFXY_HG002x1xHG002x2_19s005136-1-1_Hasenfeld_lane1HG002x02PE20403_1_sequence.txt.gz	HWVTJAFXY_HG002x1xHG002x2_19s005136-1-1_Hasenfeld_lane1HG002x02PE20403_2_sequence.txt.gz	HighQuality
HWVTJAFXY_HG002x1xHG002x2_19s005136-1-1_Hasenfeld_lane1HG002x02PE20405_1_sequence.txt.gz	HWVTJAFXY_HG002x1xHG002x2_19s005136-1-1_Hasenfeld_lane1HG002x02PE20405_2_sequence.txt.gz	HighQuality
HWVTJAFXY_HG002x1xHG002x2_19s005136-1-1_Hasenfeld_lane1HG002x02PE20407_1_sequence.txt.gz	HWVTJAFXY_HG002x1xHG002x2_19s005136-1-1_Hasenfeld_lane1HG002x02PE20407_2_sequence.txt.gz	HighQuality
HWVTJAFXY_HG002x1xHG002x2_19s005136-1-1_Hasenfeld_lane1HG002x02PE20414_1_sequence.txt.gz	HWVTJAFXY_HG002x1xHG002x2_19s005136-1-1_Hasenfeld_lane1HG002x02PE20414_2_sequence.txt.gz	HighQuality
HWVTJAFXY_HG002x1xHG002x2_19s005136-1-1_Hasenfeld_lane1HG002x02PE20416_1_sequence.txt.gz	HWVTJAFXY_HG002x1xHG002x2_19s005136-1-1_Hasenfeld_lane1HG002x02PE20416_2_sequence.txt.gz	HighQuality
HWVTJAFXY_HG002x1xHG002x2_19s005136-1-1_Hasenfeld_lane1HG002x02PE20417_1_sequence.txt.gz	HWVTJAFXY_HG002x1xHG002x2_19s005136-1-1_Hasenfeld_lane1HG002x02PE20417_2_sequence.txt.gz	HighQuality
HWVTJAFXY_HG002x1xHG002x2_19s005136-1-1_Hasenfeld_lane1HG002x02PE20419_1_sequence.txt.gz	HWVTJAFXY_HG002x1xHG002x2_19s005136-1-1_Hasenfeld_lane1HG002x02PE20419_2_sequence.txt.gz	HighQuality
HWVTJAFXY_HG002x1xHG002x2_19s005136-1-1_Hasenfeld_lane1HG002x02PE20421_1_sequence.txt.gz	HWVTJAFXY_HG002x1xHG002x2_19s005136-1-1_Hasenfeld_lane1HG002x02PE20421_2_sequence.txt.gz	HighQuality
HWVTJAFXY_HG002x1xHG002x2_19s005136-1-1_Hasenfeld_lane1HG002x02PE20422_1_sequence.txt.gz	HWVTJAFXY_HG002x1xHG002x2_19s005136-1-1_Hasenfeld_lane1HG002x02PE20422_2_sequence.txt.gz	HighQuality
HWVTJAFXY_HG002x1xHG002x2_19s005136-1-1_Hasenfeld_lane1HG002x02PE20424_1_sequence.txt.gz	HWVTJAFXY_HG002x1xHG002x2_19s005136-1-1_Hasenfeld_lane1HG002x02PE20424_2_sequence.txt.gz	HighQuality
HWVTJAFXY_HG002x1xHG002x2_19s005136-1-1_Hasenfeld_lane1HG002x02PE20425_1_sequence.txt.gz	HWVTJAFXY_HG002x1xHG002x2_19s005136-1-1_Hasenfeld_lane1HG002x02PE20425_2_sequence.txt.gz	HighQuality
HWVTJAFXY_HG002x1xHG002x2_19s005136-1-1_Hasenfeld_lane1HG002x02PE20428_1_sequence.txt.gz	HWVTJAFXY_HG002x1xHG002x2_19s005136-1-1_Hasenfeld_lane1HG002x02PE20428_2_sequence.txt.gz	HighQuality
HWVTJAFXY_HG002x1xHG002x2_19s005136-1-1_Hasenfeld_lane1HG002x02PE20430_1_sequence.txt.gz	HWVTJAFXY_HG002x1xHG002x2_19s005136-1-1_Hasenfeld_lane1HG002x02PE20430_2_sequence.txt.gz	HighQuality
HWVTJAFXY_HG002x1xHG002x2_19s005136-1-1_Hasenfeld_lane1HG002x02PE20431_1_sequence.txt.gz	HWVTJAFXY_HG002x1xHG002x2_19s005136-1-1_Hasenfeld_lane1HG002x02PE20431_2_sequence.txt.gz	HighQuality
HWVTJAFXY_HG002x1xHG002x2_19s005136-1-1_Hasenfeld_lane1HG002x02PE20432_1_sequence.txt.gz	HWVTJAFXY_HG002x1xHG002x2_19s005136-1-1_Hasenfeld_lane1HG002x02PE20432_2_sequence.txt.gz	HighQuality
HWVTJAFXY_HG002x1xHG002x2_19s005136-1-1_Hasenfeld_lane1HG002x02PE20434_1_sequence.txt.gz	HWVTJAFXY_HG002x1xHG002x2_19s005136-1-1_Hasenfeld_lane1HG002x02PE20434_2_sequence.txt.gz	HighQuality
HWVTJAFXY_HG002x1xHG002x2_19s005136-1-1_Hasenfeld_lane1HG002x02PE20435_1_sequence.txt.gz	HWVTJAFXY_HG002x1xHG002x2_19s005136-1-1_Hasenfeld_lane1HG002x02PE20435_2_sequence.txt.gz	HighQuality
HWVTJAFXY_HG002x1xHG002x2_19s005136-1-1_Hasenfeld_lane1HG002x02PE20439_1_sequence.txt.gz	HWVTJAFXY_HG002x1xHG002x2_19s005136-1-1_Hasenfeld_lane1HG002x02PE20439_2_sequence.txt.gz	HighQuality
HWVTJAFXY_HG002x1xHG002x2_19s005136-1-1_Hasenfeld_lane1HG002x02PE20440_1_sequence.txt.gz	HWVTJAFXY_HG002x1xHG002x2_19s005136-1-1_Hasenfeld_lane1HG002x02PE20440_2_sequence.txt.gz	HighQuality
HWVTJAFXY_HG002x1xHG002x2_19s005136-1-1_Hasenfeld_lane1HG002x02PE20443_1_sequence.txt.gz	HWVTJAFXY_HG002x1xHG002x2_19s005136-1-1_Hasenfeld_lane1HG002x02PE20443_2_sequence.txt.gz	HighQuality
HWVTJAFXY_HG002x1xHG002x2_19s005136-1-1_Hasenfeld_lane1HG002x02PE20445_1_sequence.txt.gz	HWVTJAFXY_HG002x1xHG002x2_19s005136-1-1_Hasenfeld_lane1HG002x02PE20445_2_sequence.txt.gz	HighQuality
HWVTJAFXY_HG002x1xHG002x2_19s005136-1-1_Hasenfeld_lane1HG002x02PE20450_1_sequence.txt.gz	HWVTJAFXY_HG002x1xHG002x2_19s005136-1-1_Hasenfeld_lane1HG002x02PE20450_2_sequence.txt.gz	HighQuality
HWVTJAFXY_HG002x1xHG002x2_19s005136-1-1_Hasenfeld_lane1HG002x02PE20452_1_sequence.txt.gz	HWVTJAFXY_HG002x1xHG002x2_19s005136-1-1_Hasenfeld_lane1HG002x02PE20452_2_sequence.txt.gz	HighQuality

HWVTJAFXY_HG002x1xHG002x2_19s005136-1-1_Hasenfeld_lane1HG002x02PE20454_1_sequence.txt.gz	HWVTJAFXY_HG002x1xHG002x2_19s005136-1-1_Hasenfeld_lane1HG002x02PE20454_2_sequence.txt.gz	HighQuality
HWVTJAFXY_HG002x1xHG002x2_19s005136-1-1_Hasenfeld_lane1HG002x02PE20456_1_sequence.txt.gz	HWVTJAFXY_HG002x1xHG002x2_19s005136-1-1_Hasenfeld_lane1HG002x02PE20456_2_sequence.txt.gz	HighQuality
HWVTJAFXY_HG002x1xHG002x2_19s005136-1-1_Hasenfeld_lane1HG002x02PE20458_1_sequence.txt.gz	HWVTJAFXY_HG002x1xHG002x2_19s005136-1-1_Hasenfeld_lane1HG002x02PE20458_2_sequence.txt.gz	HighQuality
HWVTJAFXY_HG002x1xHG002x2_19s005136-1-1_Hasenfeld_lane1HG002x02PE20464_1_sequence.txt.gz	HWVTJAFXY_HG002x1xHG002x2_19s005136-1-1_Hasenfeld_lane1HG002x02PE20464_2_sequence.txt.gz	HighQuality
HWVTJAFXY_HG002x1xHG002x2_19s005136-1-1_Hasenfeld_lane1HG002x02PE20466_1_sequence.txt.gz	HWVTJAFXY_HG002x1xHG002x2_19s005136-1-1_Hasenfeld_lane1HG002x02PE20466_2_sequence.txt.gz	HighQuality
HWVTJAFXY_HG002x1xHG002x2_19s005136-1-1_Hasenfeld_lane1HG002x02PE20467_1_sequence.txt.gz	HWVTJAFXY_HG002x1xHG002x2_19s005136-1-1_Hasenfeld_lane1HG002x02PE20467_2_sequence.txt.gz	HighQuality
HWVTJAFXY_HG002x1xHG002x2_19s005136-1-1_Hasenfeld_lane1HG002x02PE20469_1_sequence.txt.gz	HWVTJAFXY_HG002x1xHG002x2_19s005136-1-1_Hasenfeld_lane1HG002x02PE20469_2_sequence.txt.gz	HighQuality
HWVTJAFXY_HG002x1xHG002x2_19s005136-1-1_Hasenfeld_lane1HG002x02PE20473_1_sequence.txt.gz	HWVTJAFXY_HG002x1xHG002x2_19s005136-1-1_Hasenfeld_lane1HG002x02PE20473_2_sequence.txt.gz	HighQuality
HWVTJAFXY_HG002x1xHG002x2_19s005136-1-1_Hasenfeld_lane1HG002x02PE20479_1_sequence.txt.gz	HWVTJAFXY_HG002x1xHG002x2_19s005136-1-1_Hasenfeld_lane1HG002x02PE20479_2_sequence.txt.gz	HighQuality
HWVTJAFXY_HG002x1xHG002x2_19s005136-1-1_Hasenfeld_lane1HG002x02PE20483_1_sequence.txt.gz	HWVTJAFXY_HG002x1xHG002x2_19s005136-1-1_Hasenfeld_lane1HG002x02PE20483_2_sequence.txt.gz	HighQuality
HWVTJAFXY_HG002x1xHG002x2_19s005136-1-1_Hasenfeld_lane1HG002x02PE20484_1_sequence.txt.gz	HWVTJAFXY_HG002x1xHG002x2_19s005136-1-1_Hasenfeld_lane1HG002x02PE20484_2_sequence.txt.gz	HighQuality
HWVTJAFXY_HG002x1xHG002x2_19s005136-1-1_Hasenfeld_lane1HG002x02PE20485_1_sequence.txt.gz	HWVTJAFXY_HG002x1xHG002x2_19s005136-1-1_Hasenfeld_lane1HG002x02PE20485_2_sequence.txt.gz	HighQuality
HWVTJAFXY_HG002x1xHG002x2_19s005136-1-1_Hasenfeld_lane1HG002x02PE20486_1_sequence.txt.gz	HWVTJAFXY_HG002x1xHG002x2_19s005136-1-1_Hasenfeld_lane1HG002x02PE20486_2_sequence.txt.gz	HighQuality
HWVTJAFXY_HG002x1xHG002x2_19s005136-1-1_Hasenfeld_lane1HG002x02PE20487_1_sequence.txt.gz	HWVTJAFXY_HG002x1xHG002x2_19s005136-1-1_Hasenfeld_lane1HG002x02PE20487_2_sequence.txt.gz	HighQuality
HWVTJAFXY_HG002x1xHG002x2_19s005136-1-1_Hasenfeld_lane1HG002x02PE20488_1_sequence.txt.gz	HWVTJAFXY_HG002x1xHG002x2_19s005136-1-1_Hasenfeld_lane1HG002x02PE20488_2_sequence.txt.gz	HighQuality
HWVTJAFXY_HG002x1xHG002x2_19s005136-1-1_Hasenfeld_lane1HG002x02PE20490_1_sequence.txt.gz	HWVTJAFXY_HG002x1xHG002x2_19s005136-1-1_Hasenfeld_lane1HG002x02PE20490_2_sequence.txt.gz	HighQuality
HWVTJAFXY_HG002x1xHG002x2_19s005136-1-1_Hasenfeld_lane1HG002x02PE20491_1_sequence.txt.gz	HWVTJAFXY_HG002x1xHG002x2_19s005136-1-1_Hasenfeld_lane1HG002x02PE20491_2_sequence.txt.gz	HighQuality
HWVTJAFXY_HG002x1xHG002x2_19s005136-1-1_Hasenfeld_lane1HG002x02PE20492_1_sequence.txt.gz	HWVTJAFXY_HG002x1xHG002x2_19s005136-1-1_Hasenfeld_lane1HG002x02PE20492_2_sequence.txt.gz	HighQuality
HWVTJAFXY_HG002x1xHG002x2_19s005136-1-1_Hasenfeld_lane1HG002x02PE20494_1_sequence.txt.gz	HWVTJAFXY_HG002x1xHG002x2_19s005136-1-1_Hasenfeld_lane1HG002x02PE20494_2_sequence.txt.gz	HighQuality
HWVTJAFXY_HG002x1xHG002x2_19s005136-1-1_Hasenfeld_lane1HG002x01PE20302_1_sequence.txt.gz	HWVTJAFXY_HG002x1xHG002x2_19s005136-1-1_Hasenfeld_lane1HG002x01PE20302_2_sequence.txt.gz	NotHighQuality
HWVTJAFXY_HG002x1xHG002x2_19s005136-1-1_Hasenfeld_lane1HG002x01PE20304_1_sequence.txt.gz	HWVTJAFXY_HG002x1xHG002x2_19s005136-1-1_Hasenfeld_lane1HG002x01PE20304_2_sequence.txt.gz	NotHighQuality
HWVTJAFXY_HG002x1xHG002x2_19s005136-1-1_Hasenfeld_lane1HG002x01PE20309_1_sequence.txt.gz	HWVTJAFXY_HG002x1xHG002x2_19s005136-1-1_Hasenfeld_lane1HG002x01PE20309_2_sequence.txt.gz	NotHighQuality
HWVTJAFXY_HG002x1xHG002x2_19s005136-1-1_Hasenfeld_lane1HG002x01PE20310_1_sequence.txt.gz	HWVTJAFXY_HG002x1xHG002x2_19s005136-1-1_Hasenfeld_lane1HG002x01PE20310_2_sequence.txt.gz	NotHighQuality
HWVTJAFXY_HG002x1xHG002x2_19s005136-1-1_Hasenfeld_lane1HG002x01PE20311_1_sequence.txt.gz	HWVTJAFXY_HG002x1xHG002x2_19s005136-1-1_Hasenfeld_lane1HG002x01PE20311_2_sequence.txt.gz	NotHighQuality
HWVTJAFXY_HG002x1xHG002x2_19s005136-1-1_Hasenfeld_lane1HG002x01PE20312_1_sequence.txt.gz	HWVTJAFXY_HG002x1xHG002x2_19s005136-1-1_Hasenfeld_lane1HG002x01PE20312_2_sequence.txt.gz	NotHighQuality

HWVTJAFXY_HG002x1xHG002x2_19s005136-1-1_Hasenfeld_lane1HG002x01PE20314_1_sequence.txt.gz	HWVTJAFXY_HG002x1xHG002x2_19s005136-1-1_Hasenfeld_lane1HG002x01PE20314_2_sequence.txt.gz	NotHighQuality
HWVTJAFXY_HG002x1xHG002x2_19s005136-1-1_Hasenfeld_lane1HG002x01PE20316_1_sequence.txt.gz	HWVTJAFXY_HG002x1xHG002x2_19s005136-1-1_Hasenfeld_lane1HG002x01PE20316_2_sequence.txt.gz	NotHighQuality
HWVTJAFXY_HG002x1xHG002x2_19s005136-1-1_Hasenfeld_lane1HG002x01PE20317_1_sequence.txt.gz	HWVTJAFXY_HG002x1xHG002x2_19s005136-1-1_Hasenfeld_lane1HG002x01PE20317_2_sequence.txt.gz	NotHighQuality
HWVTJAFXY_HG002x1xHG002x2_19s005136-1-1_Hasenfeld_lane1HG002x01PE20320_1_sequence.txt.gz	HWVTJAFXY_HG002x1xHG002x2_19s005136-1-1_Hasenfeld_lane1HG002x01PE20320_2_sequence.txt.gz	NotHighQuality
HWVTJAFXY_HG002x1xHG002x2_19s005136-1-1_Hasenfeld_lane1HG002x01PE20321_1_sequence.txt.gz	HWVTJAFXY_HG002x1xHG002x2_19s005136-1-1_Hasenfeld_lane1HG002x01PE20321_2_sequence.txt.gz	NotHighQuality
HWVTJAFXY_HG002x1xHG002x2_19s005136-1-1_Hasenfeld_lane1HG002x01PE20322_1_sequence.txt.gz	HWVTJAFXY_HG002x1xHG002x2_19s005136-1-1_Hasenfeld_lane1HG002x01PE20322_2_sequence.txt.gz	NotHighQuality
HWVTJAFXY_HG002x1xHG002x2_19s005136-1-1_Hasenfeld_lane1HG002x01PE20323_1_sequence.txt.gz	HWVTJAFXY_HG002x1xHG002x2_19s005136-1-1_Hasenfeld_lane1HG002x01PE20323_2_sequence.txt.gz	NotHighQuality
HWVTJAFXY_HG002x1xHG002x2_19s005136-1-1_Hasenfeld_lane1HG002x01PE20324_1_sequence.txt.gz	HWVTJAFXY_HG002x1xHG002x2_19s005136-1-1_Hasenfeld_lane1HG002x01PE20324_2_sequence.txt.gz	NotHighQuality
HWVTJAFXY_HG002x1xHG002x2_19s005136-1-1_Hasenfeld_lane1HG002x01PE20326_1_sequence.txt.gz	HWVTJAFXY_HG002x1xHG002x2_19s005136-1-1_Hasenfeld_lane1HG002x01PE20326_2_sequence.txt.gz	NotHighQuality
HWVTJAFXY_HG002x1xHG002x2_19s005136-1-1_Hasenfeld_lane1HG002x01PE20330_1_sequence.txt.gz	HWVTJAFXY_HG002x1xHG002x2_19s005136-1-1_Hasenfeld_lane1HG002x01PE20330_2_sequence.txt.gz	NotHighQuality
HWVTJAFXY_HG002x1xHG002x2_19s005136-1-1_Hasenfeld_lane1HG002x01PE20333_1_sequence.txt.gz	HWVTJAFXY_HG002x1xHG002x2_19s005136-1-1_Hasenfeld_lane1HG002x01PE20333_2_sequence.txt.gz	NotHighQuality
HWVTJAFXY_HG002x1xHG002x2_19s005136-1-1_Hasenfeld_lane1HG002x01PE20338_1_sequence.txt.gz	HWVTJAFXY_HG002x1xHG002x2_19s005136-1-1_Hasenfeld_lane1HG002x01PE20338_2_sequence.txt.gz	NotHighQuality
HWVTJAFXY_HG002x1xHG002x2_19s005136-1-1_Hasenfeld_lane1HG002x01PE20344_1_sequence.txt.gz	HWVTJAFXY_HG002x1xHG002x2_19s005136-1-1_Hasenfeld_lane1HG002x01PE20344_2_sequence.txt.gz	NotHighQuality
HWVTJAFXY_HG002x1xHG002x2_19s005136-1-1_Hasenfeld_lane1HG002x01PE20346_1_sequence.txt.gz	HWVTJAFXY_HG002x1xHG002x2_19s005136-1-1_Hasenfeld_lane1HG002x01PE20346_2_sequence.txt.gz	NotHighQuality
HWVTJAFXY_HG002x1xHG002x2_19s005136-1-1_Hasenfeld_lane1HG002x01PE20348_1_sequence.txt.gz	HWVTJAFXY_HG002x1xHG002x2_19s005136-1-1_Hasenfeld_lane1HG002x01PE20348_2_sequence.txt.gz	NotHighQuality
HWVTJAFXY_HG002x1xHG002x2_19s005136-1-1_Hasenfeld_lane1HG002x01PE20349_1_sequence.txt.gz	HWVTJAFXY_HG002x1xHG002x2_19s005136-1-1_Hasenfeld_lane1HG002x01PE20349_2_sequence.txt.gz	NotHighQuality
HWVTJAFXY_HG002x1xHG002x2_19s005136-1-1_Hasenfeld_lane1HG002x01PE20354_1_sequence.txt.gz	HWVTJAFXY_HG002x1xHG002x2_19s005136-1-1_Hasenfeld_lane1HG002x01PE20354_2_sequence.txt.gz	NotHighQuality
HWVTJAFXY_HG002x1xHG002x2_19s005136-1-1_Hasenfeld_lane1HG002x01PE20360_1_sequence.txt.gz	HWVTJAFXY_HG002x1xHG002x2_19s005136-1-1_Hasenfeld_lane1HG002x01PE20360_2_sequence.txt.gz	NotHighQuality
HWVTJAFXY_HG002x1xHG002x2_19s005136-1-1_Hasenfeld_lane1HG002x01PE20365_1_sequence.txt.gz	HWVTJAFXY_HG002x1xHG002x2_19s005136-1-1_Hasenfeld_lane1HG002x01PE20365_2_sequence.txt.gz	NotHighQuality
HWVTJAFXY_HG002x1xHG002x2_19s005136-1-1_Hasenfeld_lane1HG002x01PE20366_1_sequence.txt.gz	HWVTJAFXY_HG002x1xHG002x2_19s005136-1-1_Hasenfeld_lane1HG002x01PE20366_2_sequence.txt.gz	NotHighQuality
HWVTJAFXY_HG002x1xHG002x2_19s005136-1-1_Hasenfeld_lane1HG002x01PE20369_1_sequence.txt.gz	HWVTJAFXY_HG002x1xHG002x2_19s005136-1-1_Hasenfeld_lane1HG002x01PE20369_2_sequence.txt.gz	NotHighQuality
HWVTJAFXY_HG002x1xHG002x2_19s005136-1-1_Hasenfeld_lane1HG002x01PE20370_1_sequence.txt.gz	HWVTJAFXY_HG002x1xHG002x2_19s005136-1-1_Hasenfeld_lane1HG002x01PE20370_2_sequence.txt.gz	NotHighQuality
HWVTJAFXY_HG002x1xHG002x2_19s005136-1-1_Hasenfeld_lane1HG002x01PE20371_1_sequence.txt.gz	HWVTJAFXY_HG002x1xHG002x2_19s005136-1-1_Hasenfeld_lane1HG002x01PE20371_2_sequence.txt.gz	NotHighQuality
HWVTJAFXY_HG002x1xHG002x2_19s005136-1-1_Hasenfeld_lane1HG002x01PE20372_1_sequence.txt.gz	HWVTJAFXY_HG002x1xHG002x2_19s005136-1-1_Hasenfeld_lane1HG002x01PE20372_2_sequence.txt.gz	NotHighQuality
HWVTJAFXY_HG002x1xHG002x2_19s005136-1-1_Hasenfeld_lane1HG002x01PE20373_1_sequence.txt.gz	HWVTJAFXY_HG002x1xHG002x2_19s005136-1-1_Hasenfeld_lane1HG002x01PE20373_2_sequence.txt.gz	NotHighQuality

HWVTJAFXY_HG002x1xHG002x2_19s005136-1-1_Hasenfeld_lane1HG002x01PE20375_1_sequence.txt.gz	HWVTJAFXY_HG002x1xHG002x2_19s005136-1-1_Hasenfeld_lane1HG002x01PE20375_2_sequence.txt.gz	NotHighQuality
HWVTJAFXY_HG002x1xHG002x2_19s005136-1-1_Hasenfeld_lane1HG002x01PE20380_1_sequence.txt.gz	HWVTJAFXY_HG002x1xHG002x2_19s005136-1-1_Hasenfeld_lane1HG002x01PE20380_2_sequence.txt.gz	NotHighQuality
HWVTJAFXY_HG002x1xHG002x2_19s005136-1-1_Hasenfeld_lane1HG002x01PE20382_1_sequence.txt.gz	HWVTJAFXY_HG002x1xHG002x2_19s005136-1-1_Hasenfeld_lane1HG002x01PE20382_2_sequence.txt.gz	NotHighQuality
HWVTJAFXY_HG002x1xHG002x2_19s005136-1-1_Hasenfeld_lane1HG002x01PE20383_1_sequence.txt.gz	HWVTJAFXY_HG002x1xHG002x2_19s005136-1-1_Hasenfeld_lane1HG002x01PE20383_2_sequence.txt.gz	NotHighQuality
HWVTJAFXY_HG002x1xHG002x2_19s005136-1-1_Hasenfeld_lane1HG002x01PE20384_1_sequence.txt.gz	HWVTJAFXY_HG002x1xHG002x2_19s005136-1-1_Hasenfeld_lane1HG002x01PE20384_2_sequence.txt.gz	NotHighQuality
HWVTJAFXY_HG002x1xHG002x2_19s005136-1-1_Hasenfeld_lane1HG002x01PE20385_1_sequence.txt.gz	HWVTJAFXY_HG002x1xHG002x2_19s005136-1-1_Hasenfeld_lane1HG002x01PE20385_2_sequence.txt.gz	NotHighQuality
HWVTJAFXY_HG002x1xHG002x2_19s005136-1-1_Hasenfeld_lane1HG002x01PE20386_1_sequence.txt.gz	HWVTJAFXY_HG002x1xHG002x2_19s005136-1-1_Hasenfeld_lane1HG002x01PE20386_2_sequence.txt.gz	NotHighQuality
HWVTJAFXY_HG002x1xHG002x2_19s005136-1-1_Hasenfeld_lane1HG002x01PE20390_1_sequence.txt.gz	HWVTJAFXY_HG002x1xHG002x2_19s005136-1-1_Hasenfeld_lane1HG002x01PE20390_2_sequence.txt.gz	NotHighQuality
HWVTJAFXY_HG002x1xHG002x2_19s005136-1-1_Hasenfeld_lane1HG002x01PE20394_1_sequence.txt.gz	HWVTJAFXY_HG002x1xHG002x2_19s005136-1-1_Hasenfeld_lane1HG002x01PE20394_2_sequence.txt.gz	NotHighQuality
HWVTJAFXY_HG002x1xHG002x2_19s005136-1-1_Hasenfeld_lane1HG002x01PE20395_1_sequence.txt.gz	HWVTJAFXY_HG002x1xHG002x2_19s005136-1-1_Hasenfeld_lane1HG002x01PE20395_2_sequence.txt.gz	NotHighQuality
HWVTJAFXY_HG002x1xHG002x2_19s005136-1-1_Hasenfeld_lane1HG002x01PE20396_1_sequence.txt.gz	HWVTJAFXY_HG002x1xHG002x2_19s005136-1-1_Hasenfeld_lane1HG002x01PE20396_2_sequence.txt.gz	NotHighQuality
HWVTJAFXY_HG002x1xHG002x2_19s005136-1-1_Hasenfeld_lane1HG002x02PE20401_1_sequence.txt.gz	HWVTJAFXY_HG002x1xHG002x2_19s005136-1-1_Hasenfeld_lane1HG002x02PE20401_2_sequence.txt.gz	NotHighQuality
HWVTJAFXY_HG002x1xHG002x2_19s005136-1-1_Hasenfeld_lane1HG002x02PE20402_1_sequence.txt.gz	HWVTJAFXY_HG002x1xHG002x2_19s005136-1-1_Hasenfeld_lane1HG002x02PE20402_2_sequence.txt.gz	NotHighQuality
HWVTJAFXY_HG002x1xHG002x2_19s005136-1-1_Hasenfeld_lane1HG002x02PE20404_1_sequence.txt.gz	HWVTJAFXY_HG002x1xHG002x2_19s005136-1-1_Hasenfeld_lane1HG002x02PE20404_2_sequence.txt.gz	NotHighQuality
HWVTJAFXY_HG002x1xHG002x2_19s005136-1-1_Hasenfeld_lane1HG002x02PE20406_1_sequence.txt.gz	HWVTJAFXY_HG002x1xHG002x2_19s005136-1-1_Hasenfeld_lane1HG002x02PE20406_2_sequence.txt.gz	NotHighQuality
HWVTJAFXY_HG002x1xHG002x2_19s005136-1-1_Hasenfeld_lane1HG002x02PE20408_1_sequence.txt.gz	HWVTJAFXY_HG002x1xHG002x2_19s005136-1-1_Hasenfeld_lane1HG002x02PE20408_2_sequence.txt.gz	NotHighQuality
HWVTJAFXY_HG002x1xHG002x2_19s005136-1-1_Hasenfeld_lane1HG002x02PE20409_1_sequence.txt.gz	HWVTJAFXY_HG002x1xHG002x2_19s005136-1-1_Hasenfeld_lane1HG002x02PE20409_2_sequence.txt.gz	NotHighQuality
HWVTJAFXY_HG002x1xHG002x2_19s005136-1-1_Hasenfeld_lane1HG002x02PE20410_1_sequence.txt.gz	HWVTJAFXY_HG002x1xHG002x2_19s005136-1-1_Hasenfeld_lane1HG002x02PE20410_2_sequence.txt.gz	NotHighQuality
HWVTJAFXY_HG002x1xHG002x2_19s005136-1-1_Hasenfeld_lane1HG002x02PE20411_1_sequence.txt.gz	HWVTJAFXY_HG002x1xHG002x2_19s005136-1-1_Hasenfeld_lane1HG002x02PE20411_2_sequence.txt.gz	NotHighQuality
HWVTJAFXY_HG002x1xHG002x2_19s005136-1-1_Hasenfeld_lane1HG002x02PE20412_1_sequence.txt.gz	HWVTJAFXY_HG002x1xHG002x2_19s005136-1-1_Hasenfeld_lane1HG002x02PE20412_2_sequence.txt.gz	NotHighQuality
HWVTJAFXY_HG002x1xHG002x2_19s005136-1-1_Hasenfeld_lane1HG002x02PE20413_1_sequence.txt.gz	HWVTJAFXY_HG002x1xHG002x2_19s005136-1-1_Hasenfeld_lane1HG002x02PE20413_2_sequence.txt.gz	NotHighQuality
HWVTJAFXY_HG002x1xHG002x2_19s005136-1-1_Hasenfeld_lane1HG002x02PE20415_1_sequence.txt.gz	HWVTJAFXY_HG002x1xHG002x2_19s005136-1-1_Hasenfeld_lane1HG002x02PE20415_2_sequence.txt.gz	NotHighQuality
HWVTJAFXY_HG002x1xHG002x2_19s005136-1-1_Hasenfeld_lane1HG002x02PE20418_1_sequence.txt.gz	HWVTJAFXY_HG002x1xHG002x2_19s005136-1-1_Hasenfeld_lane1HG002x02PE20418_2_sequence.txt.gz	NotHighQuality
HWVTJAFXY_HG002x1xHG002x2_19s005136-1-1_Hasenfeld_lane1HG002x02PE20420_1_sequence.txt.gz	HWVTJAFXY_HG002x1xHG002x2_19s005136-1-1_Hasenfeld_lane1HG002x02PE20420_2_sequence.txt.gz	NotHighQuality
HWVTJAFXY_HG002x1xHG002x2_19s005136-1-1_Hasenfeld_lane1HG002x02PE20423_1_sequence.txt.gz	HWVTJAFXY_HG002x1xHG002x2_19s005136-1-1_Hasenfeld_lane1HG002x02PE20423_2_sequence.txt.gz	NotHighQuality

HWVTJAFXY_HG002x1xHG002x2_19s005136-1-1_Hasenfeld_lane1HG002x02PE20426_1_sequence.txt.gz	HWVTJAFXY_HG002x1xHG002x2_19s005136-1-1_Hasenfeld_lane1HG002x02PE20426_2_sequence.txt.gz	NotHighQuality
HWVTJAFXY_HG002x1xHG002x2_19s005136-1-1_Hasenfeld_lane1HG002x02PE20427_1_sequence.txt.gz	HWVTJAFXY_HG002x1xHG002x2_19s005136-1-1_Hasenfeld_lane1HG002x02PE20427_2_sequence.txt.gz	NotHighQuality
HWVTJAFXY_HG002x1xHG002x2_19s005136-1-1_Hasenfeld_lane1HG002x02PE20429_1_sequence.txt.gz	HWVTJAFXY_HG002x1xHG002x2_19s005136-1-1_Hasenfeld_lane1HG002x02PE20429_2_sequence.txt.gz	NotHighQuality
HWVTJAFXY_HG002x1xHG002x2_19s005136-1-1_Hasenfeld_lane1HG002x02PE20433_1_sequence.txt.gz	HWVTJAFXY_HG002x1xHG002x2_19s005136-1-1_Hasenfeld_lane1HG002x02PE20433_2_sequence.txt.gz	NotHighQuality
HWVTJAFXY_HG002x1xHG002x2_19s005136-1-1_Hasenfeld_lane1HG002x02PE20436_1_sequence.txt.gz	HWVTJAFXY_HG002x1xHG002x2_19s005136-1-1_Hasenfeld_lane1HG002x02PE20436_2_sequence.txt.gz	NotHighQuality
HWVTJAFXY_HG002x1xHG002x2_19s005136-1-1_Hasenfeld_lane1HG002x02PE20437_1_sequence.txt.gz	HWVTJAFXY_HG002x1xHG002x2_19s005136-1-1_Hasenfeld_lane1HG002x02PE20437_2_sequence.txt.gz	NotHighQuality
HWVTJAFXY_HG002x1xHG002x2_19s005136-1-1_Hasenfeld_lane1HG002x02PE20438_1_sequence.txt.gz	HWVTJAFXY_HG002x1xHG002x2_19s005136-1-1_Hasenfeld_lane1HG002x02PE20438_2_sequence.txt.gz	NotHighQuality
HWVTJAFXY_HG002x1xHG002x2_19s005136-1-1_Hasenfeld_lane1HG002x02PE20441_1_sequence.txt.gz	HWVTJAFXY_HG002x1xHG002x2_19s005136-1-1_Hasenfeld_lane1HG002x02PE20441_2_sequence.txt.gz	NotHighQuality
HWVTJAFXY_HG002x1xHG002x2_19s005136-1-1_Hasenfeld_lane1HG002x02PE20442_1_sequence.txt.gz	HWVTJAFXY_HG002x1xHG002x2_19s005136-1-1_Hasenfeld_lane1HG002x02PE20442_2_sequence.txt.gz	NotHighQuality
HWVTJAFXY_HG002x1xHG002x2_19s005136-1-1_Hasenfeld_lane1HG002x02PE20444_1_sequence.txt.gz	HWVTJAFXY_HG002x1xHG002x2_19s005136-1-1_Hasenfeld_lane1HG002x02PE20444_2_sequence.txt.gz	NotHighQuality
HWVTJAFXY_HG002x1xHG002x2_19s005136-1-1_Hasenfeld_lane1HG002x02PE20446_1_sequence.txt.gz	HWVTJAFXY_HG002x1xHG002x2_19s005136-1-1_Hasenfeld_lane1HG002x02PE20446_2_sequence.txt.gz	NotHighQuality
HWVTJAFXY_HG002x1xHG002x2_19s005136-1-1_Hasenfeld_lane1HG002x02PE20447_1_sequence.txt.gz	HWVTJAFXY_HG002x1xHG002x2_19s005136-1-1_Hasenfeld_lane1HG002x02PE20447_2_sequence.txt.gz	NotHighQuality
HWVTJAFXY_HG002x1xHG002x2_19s005136-1-1_Hasenfeld_lane1HG002x02PE20448_1_sequence.txt.gz	HWVTJAFXY_HG002x1xHG002x2_19s005136-1-1_Hasenfeld_lane1HG002x02PE20448_2_sequence.txt.gz	NotHighQuality
HWVTJAFXY_HG002x1xHG002x2_19s005136-1-1_Hasenfeld_lane1HG002x02PE20449_1_sequence.txt.gz	HWVTJAFXY_HG002x1xHG002x2_19s005136-1-1_Hasenfeld_lane1HG002x02PE20449_2_sequence.txt.gz	NotHighQuality
HWVTJAFXY_HG002x1xHG002x2_19s005136-1-1_Hasenfeld_lane1HG002x02PE20451_1_sequence.txt.gz	HWVTJAFXY_HG002x1xHG002x2_19s005136-1-1_Hasenfeld_lane1HG002x02PE20451_2_sequence.txt.gz	NotHighQuality
HWVTJAFXY_HG002x1xHG002x2_19s005136-1-1_Hasenfeld_lane1HG002x02PE20453_1_sequence.txt.gz	HWVTJAFXY_HG002x1xHG002x2_19s005136-1-1_Hasenfeld_lane1HG002x02PE20453_2_sequence.txt.gz	NotHighQuality
HWVTJAFXY_HG002x1xHG002x2_19s005136-1-1_Hasenfeld_lane1HG002x02PE20455_1_sequence.txt.gz	HWVTJAFXY_HG002x1xHG002x2_19s005136-1-1_Hasenfeld_lane1HG002x02PE20455_2_sequence.txt.gz	NotHighQuality
HWVTJAFXY_HG002x1xHG002x2_19s005136-1-1_Hasenfeld_lane1HG002x02PE20457_1_sequence.txt.gz	HWVTJAFXY_HG002x1xHG002x2_19s005136-1-1_Hasenfeld_lane1HG002x02PE20457_2_sequence.txt.gz	NotHighQuality
HWVTJAFXY_HG002x1xHG002x2_19s005136-1-1_Hasenfeld_lane1HG002x02PE20459_1_sequence.txt.gz	HWVTJAFXY_HG002x1xHG002x2_19s005136-1-1_Hasenfeld_lane1HG002x02PE20459_2_sequence.txt.gz	NotHighQuality
HWVTJAFXY_HG002x1xHG002x2_19s005136-1-1_Hasenfeld_lane1HG002x02PE20460_1_sequence.txt.gz	HWVTJAFXY_HG002x1xHG002x2_19s005136-1-1_Hasenfeld_lane1HG002x02PE20460_2_sequence.txt.gz	NotHighQuality
HWVTJAFXY_HG002x1xHG002x2_19s005136-1-1_Hasenfeld_lane1HG002x02PE20461_1_sequence.txt.gz	HWVTJAFXY_HG002x1xHG002x2_19s005136-1-1_Hasenfeld_lane1HG002x02PE20461_2_sequence.txt.gz	NotHighQuality
HWVTJAFXY_HG002x1xHG002x2_19s005136-1-1_Hasenfeld_lane1HG002x02PE20462_1_sequence.txt.gz	HWVTJAFXY_HG002x1xHG002x2_19s005136-1-1_Hasenfeld_lane1HG002x02PE20462_2_sequence.txt.gz	NotHighQuality
HWVTJAFXY_HG002x1xHG002x2_19s005136-1-1_Hasenfeld_lane1HG002x02PE20463_1_sequence.txt.gz	HWVTJAFXY_HG002x1xHG002x2_19s005136-1-1_Hasenfeld_lane1HG002x02PE20463_2_sequence.txt.gz	NotHighQuality
HWVTJAFXY_HG002x1xHG002x2_19s005136-1-1_Hasenfeld_lane1HG002x02PE20465_1_sequence.txt.gz	HWVTJAFXY_HG002x1xHG002x2_19s005136-1-1_Hasenfeld_lane1HG002x02PE20465_2_sequence.txt.gz	NotHighQuality
HWVTJAFXY_HG002x1xHG002x2_19s005136-1-1_Hasenfeld_lane1HG002x02PE20468_1_sequence.txt.gz	HWVTJAFXY_HG002x1xHG002x2_19s005136-1-1_Hasenfeld_lane1HG002x02PE20468_2_sequence.txt.gz	NotHighQuality

HWVTJAFXY_HG002x1xHG002x2_19s005136-1-1_Hasenfeld_lane1HG002x02PE20470_1_sequence.txt.gz	HWVTJAFXY_HG002x1xHG002x2_19s005136-1-1_Hasenfeld_lane1HG002x02PE20470_2_sequence.txt.gz	NotHighQuality
HWVTJAFXY_HG002x1xHG002x2_19s005136-1-1_Hasenfeld_lane1HG002x02PE20471_1_sequence.txt.gz	HWVTJAFXY_HG002x1xHG002x2_19s005136-1-1_Hasenfeld_lane1HG002x02PE20471_2_sequence.txt.gz	NotHighQuality
HWVTJAFXY_HG002x1xHG002x2_19s005136-1-1_Hasenfeld_lane1HG002x02PE20472_1_sequence.txt.gz	HWVTJAFXY_HG002x1xHG002x2_19s005136-1-1_Hasenfeld_lane1HG002x02PE20472_2_sequence.txt.gz	NotHighQuality
HWVTJAFXY_HG002x1xHG002x2_19s005136-1-1_Hasenfeld_lane1HG002x02PE20474_1_sequence.txt.gz	HWVTJAFXY_HG002x1xHG002x2_19s005136-1-1_Hasenfeld_lane1HG002x02PE20474_2_sequence.txt.gz	NotHighQuality
HWVTJAFXY_HG002x1xHG002x2_19s005136-1-1_Hasenfeld_lane1HG002x02PE20475_1_sequence.txt.gz	HWVTJAFXY_HG002x1xHG002x2_19s005136-1-1_Hasenfeld_lane1HG002x02PE20475_2_sequence.txt.gz	NotHighQuality
HWVTJAFXY_HG002x1xHG002x2_19s005136-1-1_Hasenfeld_lane1HG002x02PE20476_1_sequence.txt.gz	HWVTJAFXY_HG002x1xHG002x2_19s005136-1-1_Hasenfeld_lane1HG002x02PE20476_2_sequence.txt.gz	NotHighQuality
HWVTJAFXY_HG002x1xHG002x2_19s005136-1-1_Hasenfeld_lane1HG002x02PE20477_1_sequence.txt.gz	HWVTJAFXY_HG002x1xHG002x2_19s005136-1-1_Hasenfeld_lane1HG002x02PE20477_2_sequence.txt.gz	NotHighQuality
HWVTJAFXY_HG002x1xHG002x2_19s005136-1-1_Hasenfeld_lane1HG002x02PE20478_1_sequence.txt.gz	HWVTJAFXY_HG002x1xHG002x2_19s005136-1-1_Hasenfeld_lane1HG002x02PE20478_2_sequence.txt.gz	NotHighQuality
HWVTJAFXY_HG002x1xHG002x2_19s005136-1-1_Hasenfeld_lane1HG002x02PE20480_1_sequence.txt.gz	HWVTJAFXY_HG002x1xHG002x2_19s005136-1-1_Hasenfeld_lane1HG002x02PE20480_2_sequence.txt.gz	NotHighQuality
HWVTJAFXY_HG002x1xHG002x2_19s005136-1-1_Hasenfeld_lane1HG002x02PE20481_1_sequence.txt.gz	HWVTJAFXY_HG002x1xHG002x2_19s005136-1-1_Hasenfeld_lane1HG002x02PE20481_2_sequence.txt.gz	NotHighQuality
HWVTJAFXY_HG002x1xHG002x2_19s005136-1-1_Hasenfeld_lane1HG002x02PE20482_1_sequence.txt.gz	HWVTJAFXY_HG002x1xHG002x2_19s005136-1-1_Hasenfeld_lane1HG002x02PE20482_2_sequence.txt.gz	NotHighQuality
HWVTJAFXY_HG002x1xHG002x2_19s005136-1-1_Hasenfeld_lane1HG002x02PE20489_1_sequence.txt.gz	HWVTJAFXY_HG002x1xHG002x2_19s005136-1-1_Hasenfeld_lane1HG002x02PE20489_2_sequence.txt.gz	NotHighQuality
HWVTJAFXY_HG002x1xHG002x2_19s005136-1-1_Hasenfeld_lane1HG002x02PE20493_1_sequence.txt.gz	HWVTJAFXY_HG002x1xHG002x2_19s005136-1-1_Hasenfeld_lane1HG002x02PE20493_2_sequence.txt.gz	NotHighQuality
HWVTJAFXY_HG002x1xHG002x2_19s005136-1-1_Hasenfeld_lane1HG002x02PE20495_1_sequence.txt.gz	HWVTJAFXY_HG002x1xHG002x2_19s005136-1-1_Hasenfeld_lane1HG002x02PE20495_2_sequence.txt.gz	NotHighQuality
HWVTJAFXY_HG002x1xHG002x2_19s005136-1-1_Hasenfeld_lane1HG002x02PE20496_1_sequence.txt.gz	HWVTJAFXY_HG002x1xHG002x2_19s005136-1-1_Hasenfeld_lane1HG002x02PE20496_2_sequence.txt.gz	NotHighQuality

Table S3: Verkko NA24385 Titration Scaffold auN

% High-Quality Libraries	Repetition	Scaffold auN (Mbp hpc)	Fraction of 192 Library Reference auN (%)
0	1	86.9	86
25	1	94.3	93
25	2	97.8	96
25	3	85.2	84
25	4	94.4	93
50	1	95.4	94
50	2	96.8	95
50	3	99.6	98
50	4	95.4	94
75	1	99.9	98

75	2	99.6	98
75	3	97.8	96
75	4	97.8	96
100	1	99.9	98

Table S4: NA24385 hifiasm HiFi Only (44.5x) Phased Assembly Evaluation Statistics

**Evaluation**	**Metric**	**Graphasing**	**Trio**
Contiguity	auN (Mbp)	66.0	67.9
Contiguity	N50 (Mbp)	57.8	64.9
yak trioeval	Switch Error Rate (%)	0.23	0.16
yak trioeval	Hamming Error Rate (%)	0.57	0.15
yak qv	QV	56.1	55.8
paftools misjoin	Gaps [acrocentric-only] (n)	7 [0]	2 [0]
paftools misjoin	Interchromsomal Misjoins [acrocentric-only] (n)	0 [3]	0 [4]
paftools misjoin	Inversions [acrocentric-only] (n)	1 [0]	0 [0]
paftools asmgene	MMC (%)	12.0	1.4
paftools asmgene	MSC (%)	1.4	0.2
