# Supplementary material for: Phasing Diploid Genome Assembly Graphs with Single-Cell Strand Sequencing
Source: bioRxiv. 2024 Feb 16:2024.02.15.580432. Preprint. [Version 1] doi: 10.1101/2024.02.15.580432 (PMC10962706; doi:10.1101/2024.02.15.580432)
Supplement: Supplementary file 1 [file media-1.pdf]

Supplement

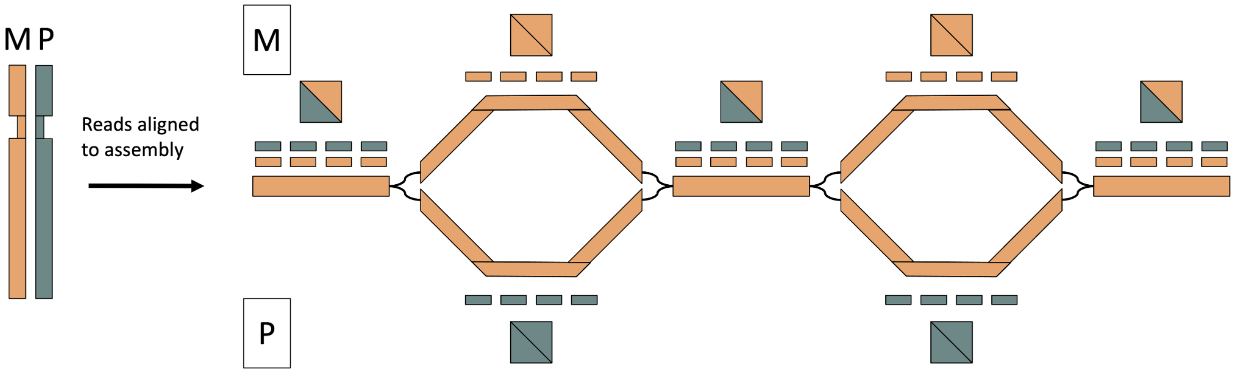

Figure S1. Strand-seq Phasing Signal. A reads from a WC Strand-seq library (left) are aligned to the unitigs of a chromosome (right). Orange shapes correspond to Watson orientation, and green shapes correspond to Crick orientation. The graph unitigs and maternal reads are all in Watson orientation, while the paternal reads are all in Crick orientation. Consequently, sequence unique to the maternal haplotype will receive only "forward" alignments (orange box), while sequence unique to the paternal haplotype will receive only "reverse" alignments (green box). Sequence shared between the haplotypes receives a 50/50 mix of forward and reverse alignments (green\orange box)

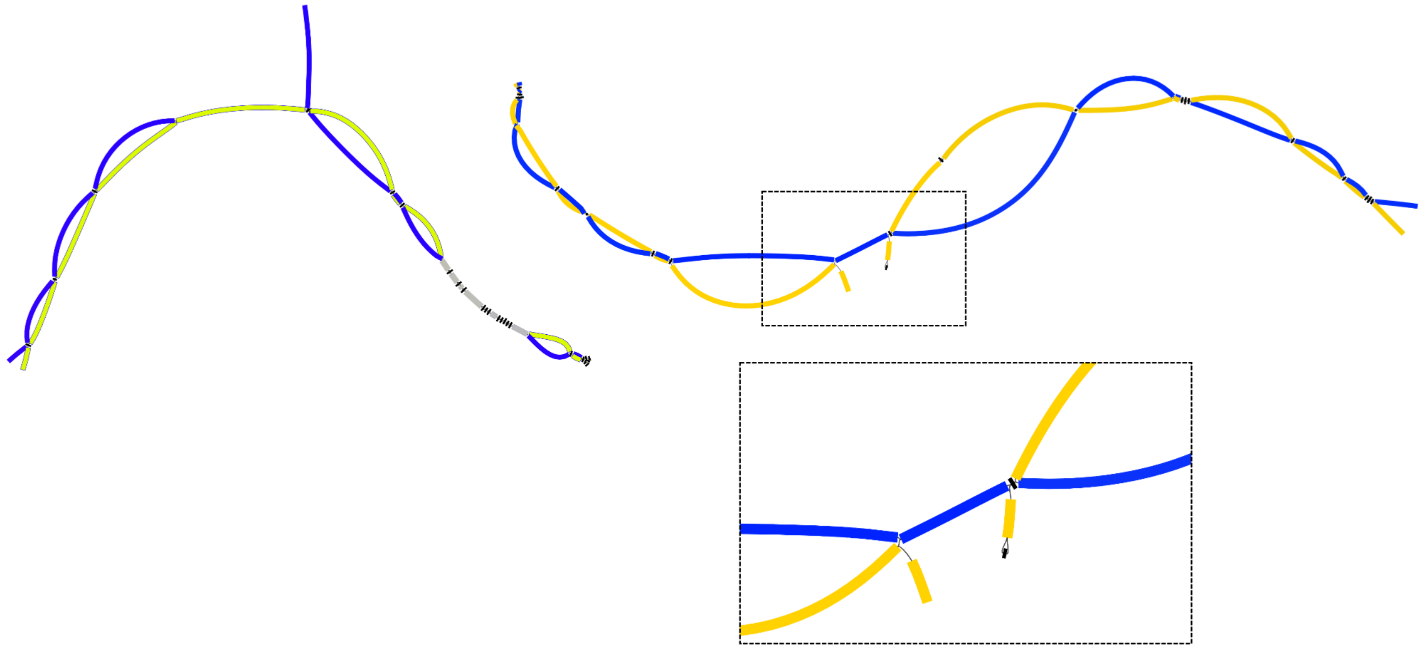

Figure S2. Hairpin-Capped Broken Bubbles interrupt Rukki Scaffolding. Bandage [67] *graphs of chromosomes 5 (left) and 1 (right, top) from the Verkko HG00733 assembly both show broken bubbles. On each connected component, the blue or yellow shade corresponds to a haplotype as inferred by Graphasing, grey corresponds to homozygous nodes, and black to nodes without inferred phasing signal. The blue haplotype on chromosome 5 is scaffolded in one path, but the yellow haplotype of chromosome 1 is represented with two scaffolds, one on*

either side of the broken bubble, and is the only pictured haplotype not connected in a single path. Zooming in (right, bottom) shows that one end of the broken bubble is "capped" by a single small node in a hairpin structure.

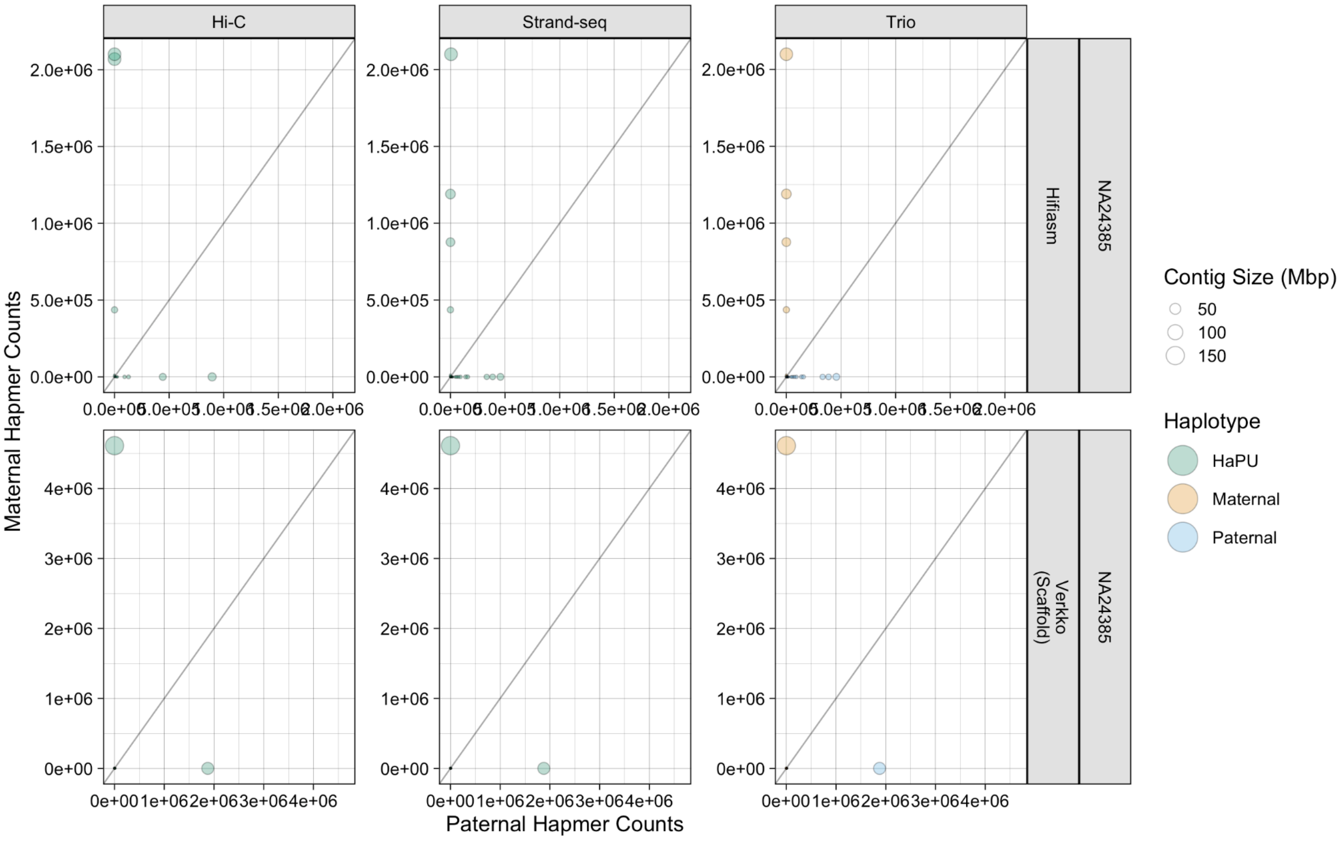

Figure S3. Sex Chromosome Hap-mer Blob Plots. NA24385 contigs aligning to the X and Y chromosomes are plotted.

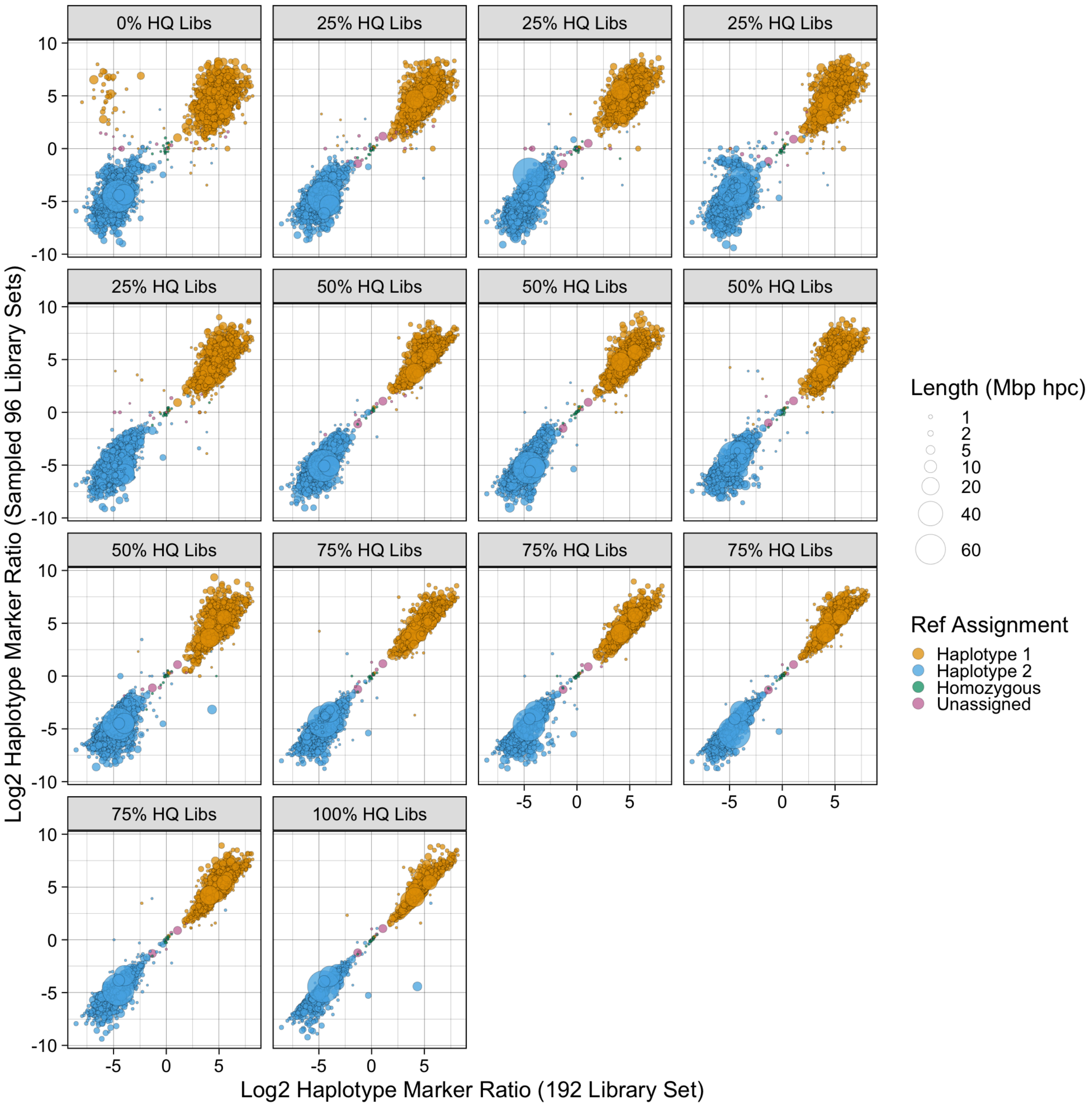

Figure S4. Marker ratio scatter plots. The haplotype markers for each sampled library set is compared to the reference set generated using all 192 available Strand-seq libraries. Each unitig is displayed as a point, with size representing size and color representing reference Rukki assignment. X and Y coordinates are the log2 of the ratio of haplotype 1 markers to haplotype 2 markers. Points close to the positive diagonal are more consistent with the reference, while those in the negative diagonal quadrants represent switch errors relative to the reference. Note that the unitigs sizes represented homopolymer compressed lengths, as the Verkko pipeline constructs its graph with homopolymer compressed sequences.

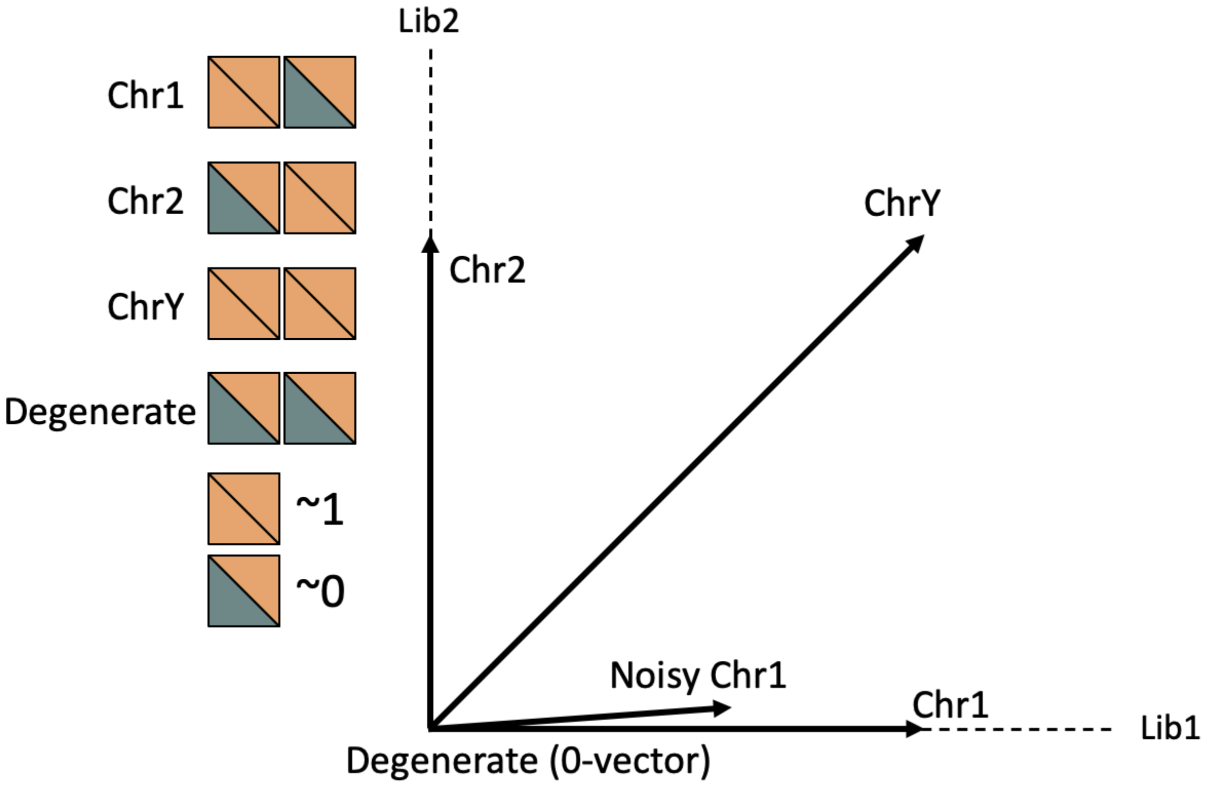

Figure S5. Behavior of Absolute SSF Calculated with bwa mem alignments. Each axis corresponds to a different Strand-seq library. A chromosome that has a homozygous strand state for a library has a value of 1 for that component, and a value of 0 if the library is heterozygous strand state. The Y chromosome, being haploid, has a homozygous strand state for all libraries, while degenerate sequence appears to have a heterozygous strand state for all libraries. The absolute SSF vector for each chromosome points to a different corner of the positive orthant, while the degenerate unitig vector is the 0 vector. A unitig that contains noisy degenerate sequence is skewed and shrunk towards the 0 vector, but the nearest corner pointed to still corresponds to the correct chromosome.

	Hap 1	Hap 2
WC Library 1	Watson	Crick
WC Library 2	Crick	Watson
WC Library 3	Crick	Watson
WC Library 4	Watson	Crick

	Hap 1	Hap 2
WC Library 1	Crick	Watson
WC Library 2	Crick	Watson
WC Library 3	Crick	Watson
WC Library 4	Crick	Watson

Figure S6. Read Labeling for Proper Strand-seq Library Pooling. In each Strand-seq library, the labels (Watson, Crick) of the reads aligning to each haplotype depend on the orientation of the unitigs and the inherited Strand-state of the heterozygous strand state library (WC vs. CW library). However, swapping the labels of reads does not affect the steps before library pooling, and so the labeling can safely be treated as if it were arbitrary. Left: Each haplotype receives only Watson or only Crick alignments within each library. However, the pooled library assigns each haplotype a 50/50 ratio of Watson and Crick alignments and fails to reflect the behavior at the individual library level. Right: The labels of libraries 1 and 4 are swapped, and the pooled library now also assigns reads of only one label to each haplotype.

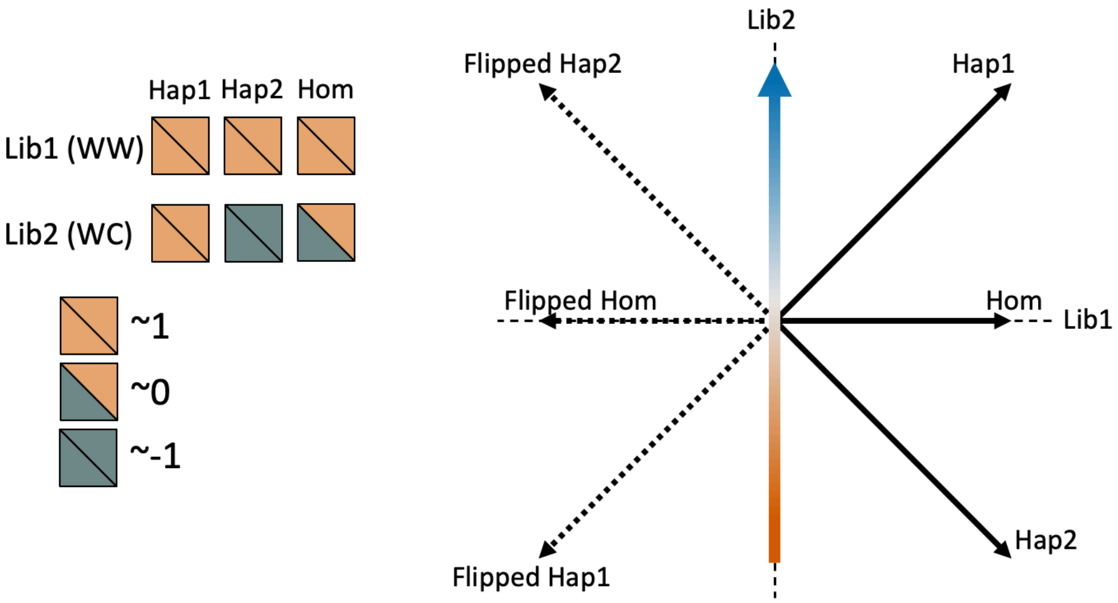

Figure S7. Self Supervised Model for WC Library Identification and Pooling. When the SSF is calculated with phase-informative reads, the haplotypes separate along the axes corresponding to homozygous strand state libraries, with homozygous nodes lying along the line between them. When a model is trained to classify unitigs and their “flipped” versions, the classification boundary (red and blue arrow) has the special property that each dimension that inherited a heterozygous strand state has the same magnitude, while each dimension that inherited a homozygous strand state has a value of 0.

Table S1: NA24385 Strand-seq library quality annotations:

File 1	File 2	Annotation
HWVTJAFXY_HG002x1xHG002x2_19s005136-1-1_Hasenfeld_lane1HG002x01PE20301_1_sequence.txt.gz	HWVTJAFXY_HG002x1xHG002x2_19s005136-1-1_Hasenfeld_lane1HG002x01PE20301_2_sequence.txt.gz	HighQuality
HWVTJAFXY_HG002x1xHG002x2_19s005136-1-1_Hasenfeld_lane1HG002x01PE20303_1_sequence.txt.gz	HWVTJAFXY_HG002x1xHG002x2_19s005136-1-1_Hasenfeld_lane1HG002x01PE20303_2_sequence.txt.gz	HighQuality
HWVTJAFXY_HG002x1xHG002x2_19s005136-1-1_Hasenfeld_lane1HG002x01PE20305_1_sequence.txt.gz	HWVTJAFXY_HG002x1xHG002x2_19s005136-1-1_Hasenfeld_lane1HG002x01PE20305_2_sequence.txt.gz	HighQuality
HWVTJAFXY_HG002x1xHG002x2_19s005136-1-1_Hasenfeld_lane1HG002x01PE20306_1_sequence.txt.gz	HWVTJAFXY_HG002x1xHG002x2_19s005136-1-1_Hasenfeld_lane1HG002x01PE20306_2_sequence.txt.gz	HighQuality
HWVTJAFXY_HG002x1xHG002x2_19s005136-1-1_Hasenfeld_lane1HG002x01PE20307_1_sequence.txt.gz	HWVTJAFXY_HG002x1xHG002x2_19s005136-1-1_Hasenfeld_lane1HG002x01PE20307_2_sequence.txt.gz	HighQuality
HWVTJAFXY_HG002x1xHG002x2_19s005136-1-1_Hasenfeld_lane1HG002x01PE20308_1_sequence.txt.gz	HWVTJAFXY_HG002x1xHG002x2_19s005136-1-1_Hasenfeld_lane1HG002x01PE20308_2_sequence.txt.gz	HighQuality
HWVTJAFXY_HG002x1xHG002x2_19s005136-1-1_Hasenfeld_lane1HG002x01PE20313_1_sequence.txt.gz	HWVTJAFXY_HG002x1xHG002x2_19s005136-1-1_Hasenfeld_lane1HG002x01PE20313_2_sequence.txt.gz	HighQuality
HWVTJAFXY_HG002x1xHG002x2_19s005136-1-1_Hasenfeld_lane1HG002x01PE20315_1_sequence.txt.gz	HWVTJAFXY_HG002x1xHG002x2_19s005136-1-1_Hasenfeld_lane1HG002x01PE20315_2_sequence.txt.gz	HighQuality
HWVTJAFXY_HG002x1xHG002x2_19s005136-1-1_Hasenfeld_lane1HG002x01PE20318_1_sequence.txt.gz	HWVTJAFXY_HG002x1xHG002x2_19s005136-1-1_Hasenfeld_lane1HG002x01PE20318_2_sequence.txt.gz	HighQuality

HWVTJAFXY_HG002x1xHG002x2_19s005136-1-1_Hasenfeld_lane1HG002x01PE20319_1_sequence.txt.gz	HWVTJAFXY_HG002x1xHG002x2_19s005136-1-1_Hasenfeld_lane1HG002x01PE20319_2_sequence.txt.gz	HighQuality
HWVTJAFXY_HG002x1xHG002x2_19s005136-1-1_Hasenfeld_lane1HG002x01PE20325_1_sequence.txt.gz	HWVTJAFXY_HG002x1xHG002x2_19s005136-1-1_Hasenfeld_lane1HG002x01PE20325_2_sequence.txt.gz	HighQuality
HWVTJAFXY_HG002x1xHG002x2_19s005136-1-1_Hasenfeld_lane1HG002x01PE20327_1_sequence.txt.gz	HWVTJAFXY_HG002x1xHG002x2_19s005136-1-1_Hasenfeld_lane1HG002x01PE20327_2_sequence.txt.gz	HighQuality
HWVTJAFXY_HG002x1xHG002x2_19s005136-1-1_Hasenfeld_lane1HG002x01PE20328_1_sequence.txt.gz	HWVTJAFXY_HG002x1xHG002x2_19s005136-1-1_Hasenfeld_lane1HG002x01PE20328_2_sequence.txt.gz	HighQuality
HWVTJAFXY_HG002x1xHG002x2_19s005136-1-1_Hasenfeld_lane1HG002x01PE20329_1_sequence.txt.gz	HWVTJAFXY_HG002x1xHG002x2_19s005136-1-1_Hasenfeld_lane1HG002x01PE20329_2_sequence.txt.gz	HighQuality
HWVTJAFXY_HG002x1xHG002x2_19s005136-1-1_Hasenfeld_lane1HG002x01PE20331_1_sequence.txt.gz	HWVTJAFXY_HG002x1xHG002x2_19s005136-1-1_Hasenfeld_lane1HG002x01PE20331_2_sequence.txt.gz	HighQuality
HWVTJAFXY_HG002x1xHG002x2_19s005136-1-1_Hasenfeld_lane1HG002x01PE20332_1_sequence.txt.gz	HWVTJAFXY_HG002x1xHG002x2_19s005136-1-1_Hasenfeld_lane1HG002x01PE20332_2_sequence.txt.gz	HighQuality
HWVTJAFXY_HG002x1xHG002x2_19s005136-1-1_Hasenfeld_lane1HG002x01PE20334_1_sequence.txt.gz	HWVTJAFXY_HG002x1xHG002x2_19s005136-1-1_Hasenfeld_lane1HG002x01PE20334_2_sequence.txt.gz	HighQuality
HWVTJAFXY_HG002x1xHG002x2_19s005136-1-1_Hasenfeld_lane1HG002x01PE20335_1_sequence.txt.gz	HWVTJAFXY_HG002x1xHG002x2_19s005136-1-1_Hasenfeld_lane1HG002x01PE20335_2_sequence.txt.gz	HighQuality
HWVTJAFXY_HG002x1xHG002x2_19s005136-1-1_Hasenfeld_lane1HG002x01PE20336_1_sequence.txt.gz	HWVTJAFXY_HG002x1xHG002x2_19s005136-1-1_Hasenfeld_lane1HG002x01PE20336_2_sequence.txt.gz	HighQuality
HWVTJAFXY_HG002x1xHG002x2_19s005136-1-1_Hasenfeld_lane1HG002x01PE20337_1_sequence.txt.gz	HWVTJAFXY_HG002x1xHG002x2_19s005136-1-1_Hasenfeld_lane1HG002x01PE20337_2_sequence.txt.gz	HighQuality
HWVTJAFXY_HG002x1xHG002x2_19s005136-1-1_Hasenfeld_lane1HG002x01PE20339_1_sequence.txt.gz	HWVTJAFXY_HG002x1xHG002x2_19s005136-1-1_Hasenfeld_lane1HG002x01PE20339_2_sequence.txt.gz	HighQuality
HWVTJAFXY_HG002x1xHG002x2_19s005136-1-1_Hasenfeld_lane1HG002x01PE20340_1_sequence.txt.gz	HWVTJAFXY_HG002x1xHG002x2_19s005136-1-1_Hasenfeld_lane1HG002x01PE20340_2_sequence.txt.gz	HighQuality
HWVTJAFXY_HG002x1xHG002x2_19s005136-1-1_Hasenfeld_lane1HG002x01PE20341_1_sequence.txt.gz	HWVTJAFXY_HG002x1xHG002x2_19s005136-1-1_Hasenfeld_lane1HG002x01PE20341_2_sequence.txt.gz	HighQuality
HWVTJAFXY_HG002x1xHG002x2_19s005136-1-1_Hasenfeld_lane1HG002x01PE20342_1_sequence.txt.gz	HWVTJAFXY_HG002x1xHG002x2_19s005136-1-1_Hasenfeld_lane1HG002x01PE20342_2_sequence.txt.gz	HighQuality
HWVTJAFXY_HG002x1xHG002x2_19s005136-1-1_Hasenfeld_lane1HG002x01PE20343_1_sequence.txt.gz	HWVTJAFXY_HG002x1xHG002x2_19s005136-1-1_Hasenfeld_lane1HG002x01PE20343_2_sequence.txt.gz	HighQuality
HWVTJAFXY_HG002x1xHG002x2_19s005136-1-1_Hasenfeld_lane1HG002x01PE20345_1_sequence.txt.gz	HWVTJAFXY_HG002x1xHG002x2_19s005136-1-1_Hasenfeld_lane1HG002x01PE20345_2_sequence.txt.gz	HighQuality
HWVTJAFXY_HG002x1xHG002x2_19s005136-1-1_Hasenfeld_lane1HG002x01PE20347_1_sequence.txt.gz	HWVTJAFXY_HG002x1xHG002x2_19s005136-1-1_Hasenfeld_lane1HG002x01PE20347_2_sequence.txt.gz	HighQuality
HWVTJAFXY_HG002x1xHG002x2_19s005136-1-1_Hasenfeld_lane1HG002x01PE20350_1_sequence.txt.gz	HWVTJAFXY_HG002x1xHG002x2_19s005136-1-1_Hasenfeld_lane1HG002x01PE20350_2_sequence.txt.gz	HighQuality
HWVTJAFXY_HG002x1xHG002x2_19s005136-1-1_Hasenfeld_lane1HG002x01PE20351_1_sequence.txt.gz	HWVTJAFXY_HG002x1xHG002x2_19s005136-1-1_Hasenfeld_lane1HG002x01PE20351_2_sequence.txt.gz	HighQuality
HWVTJAFXY_HG002x1xHG002x2_19s005136-1-1_Hasenfeld_lane1HG002x01PE20352_1_sequence.txt.gz	HWVTJAFXY_HG002x1xHG002x2_19s005136-1-1_Hasenfeld_lane1HG002x01PE20352_2_sequence.txt.gz	HighQuality
HWVTJAFXY_HG002x1xHG002x2_19s005136-1-1_Hasenfeld_lane1HG002x01PE20353_1_sequence.txt.gz	HWVTJAFXY_HG002x1xHG002x2_19s005136-1-1_Hasenfeld_lane1HG002x01PE20353_2_sequence.txt.gz	HighQuality
HWVTJAFXY_HG002x1xHG002x2_19s005136-1-1_Hasenfeld_lane1HG002x01PE20355_1_sequence.txt.gz	HWVTJAFXY_HG002x1xHG002x2_19s005136-1-1_Hasenfeld_lane1HG002x01PE20355_2_sequence.txt.gz	HighQuality
HWVTJAFXY_HG002x1xHG002x2_19s005136-1-1_Hasenfeld_lane1HG002x01PE20356_1_sequence.txt.gz	HWVTJAFXY_HG002x1xHG002x2_19s005136-1-1_Hasenfeld_lane1HG002x01PE20356_2_sequence.txt.gz	HighQuality
HWVTJAFXY_HG002x1xHG002x2_19s005136-1-1_Hasenfeld_lane1HG002x01PE20357_1_sequence.txt.gz	HWVTJAFXY_HG002x1xHG002x2_19s005136-1-1_Hasenfeld_lane1HG002x01PE20357_2_sequence.txt.gz	HighQuality

HWVTJAFXY_HG002x1xHG002x2_19s005136-1-1_Hasenfeld_lane1HG002x01PE20358_1_sequence.txt.gz	HWVTJAFXY_HG002x1xHG002x2_19s005136-1-1_Hasenfeld_lane1HG002x01PE20358_2_sequence.txt.gz	HighQuality
HWVTJAFXY_HG002x1xHG002x2_19s005136-1-1_Hasenfeld_lane1HG002x01PE20359_1_sequence.txt.gz	HWVTJAFXY_HG002x1xHG002x2_19s005136-1-1_Hasenfeld_lane1HG002x01PE20359_2_sequence.txt.gz	HighQuality
HWVTJAFXY_HG002x1xHG002x2_19s005136-1-1_Hasenfeld_lane1HG002x01PE20361_1_sequence.txt.gz	HWVTJAFXY_HG002x1xHG002x2_19s005136-1-1_Hasenfeld_lane1HG002x01PE20361_2_sequence.txt.gz	HighQuality
HWVTJAFXY_HG002x1xHG002x2_19s005136-1-1_Hasenfeld_lane1HG002x01PE20362_1_sequence.txt.gz	HWVTJAFXY_HG002x1xHG002x2_19s005136-1-1_Hasenfeld_lane1HG002x01PE20362_2_sequence.txt.gz	HighQuality
HWVTJAFXY_HG002x1xHG002x2_19s005136-1-1_Hasenfeld_lane1HG002x01PE20363_1_sequence.txt.gz	HWVTJAFXY_HG002x1xHG002x2_19s005136-1-1_Hasenfeld_lane1HG002x01PE20363_2_sequence.txt.gz	HighQuality
HWVTJAFXY_HG002x1xHG002x2_19s005136-1-1_Hasenfeld_lane1HG002x01PE20364_1_sequence.txt.gz	HWVTJAFXY_HG002x1xHG002x2_19s005136-1-1_Hasenfeld_lane1HG002x01PE20364_2_sequence.txt.gz	HighQuality
HWVTJAFXY_HG002x1xHG002x2_19s005136-1-1_Hasenfeld_lane1HG002x01PE20367_1_sequence.txt.gz	HWVTJAFXY_HG002x1xHG002x2_19s005136-1-1_Hasenfeld_lane1HG002x01PE20367_2_sequence.txt.gz	HighQuality
HWVTJAFXY_HG002x1xHG002x2_19s005136-1-1_Hasenfeld_lane1HG002x01PE20368_1_sequence.txt.gz	HWVTJAFXY_HG002x1xHG002x2_19s005136-1-1_Hasenfeld_lane1HG002x01PE20368_2_sequence.txt.gz	HighQuality
HWVTJAFXY_HG002x1xHG002x2_19s005136-1-1_Hasenfeld_lane1HG002x01PE20374_1_sequence.txt.gz	HWVTJAFXY_HG002x1xHG002x2_19s005136-1-1_Hasenfeld_lane1HG002x01PE20374_2_sequence.txt.gz	HighQuality
HWVTJAFXY_HG002x1xHG002x2_19s005136-1-1_Hasenfeld_lane1HG002x01PE20376_1_sequence.txt.gz	HWVTJAFXY_HG002x1xHG002x2_19s005136-1-1_Hasenfeld_lane1HG002x01PE20376_2_sequence.txt.gz	HighQuality
HWVTJAFXY_HG002x1xHG002x2_19s005136-1-1_Hasenfeld_lane1HG002x01PE20377_1_sequence.txt.gz	HWVTJAFXY_HG002x1xHG002x2_19s005136-1-1_Hasenfeld_lane1HG002x01PE20377_2_sequence.txt.gz	HighQuality
HWVTJAFXY_HG002x1xHG002x2_19s005136-1-1_Hasenfeld_lane1HG002x01PE20378_1_sequence.txt.gz	HWVTJAFXY_HG002x1xHG002x2_19s005136-1-1_Hasenfeld_lane1HG002x01PE20378_2_sequence.txt.gz	HighQuality
HWVTJAFXY_HG002x1xHG002x2_19s005136-1-1_Hasenfeld_lane1HG002x01PE20379_1_sequence.txt.gz	HWVTJAFXY_HG002x1xHG002x2_19s005136-1-1_Hasenfeld_lane1HG002x01PE20379_2_sequence.txt.gz	HighQuality
HWVTJAFXY_HG002x1xHG002x2_19s005136-1-1_Hasenfeld_lane1HG002x01PE20381_1_sequence.txt.gz	HWVTJAFXY_HG002x1xHG002x2_19s005136-1-1_Hasenfeld_lane1HG002x01PE20381_2_sequence.txt.gz	HighQuality
HWVTJAFXY_HG002x1xHG002x2_19s005136-1-1_Hasenfeld_lane1HG002x01PE20387_1_sequence.txt.gz	HWVTJAFXY_HG002x1xHG002x2_19s005136-1-1_Hasenfeld_lane1HG002x01PE20387_2_sequence.txt.gz	HighQuality
HWVTJAFXY_HG002x1xHG002x2_19s005136-1-1_Hasenfeld_lane1HG002x01PE20388_1_sequence.txt.gz	HWVTJAFXY_HG002x1xHG002x2_19s005136-1-1_Hasenfeld_lane1HG002x01PE20388_2_sequence.txt.gz	HighQuality
HWVTJAFXY_HG002x1xHG002x2_19s005136-1-1_Hasenfeld_lane1HG002x01PE20389_1_sequence.txt.gz	HWVTJAFXY_HG002x1xHG002x2_19s005136-1-1_Hasenfeld_lane1HG002x01PE20389_2_sequence.txt.gz	HighQuality
HWVTJAFXY_HG002x1xHG002x2_19s005136-1-1_Hasenfeld_lane1HG002x01PE20391_1_sequence.txt.gz	HWVTJAFXY_HG002x1xHG002x2_19s005136-1-1_Hasenfeld_lane1HG002x01PE20391_2_sequence.txt.gz	HighQuality
HWVTJAFXY_HG002x1xHG002x2_19s005136-1-1_Hasenfeld_lane1HG002x01PE20392_1_sequence.txt.gz	HWVTJAFXY_HG002x1xHG002x2_19s005136-1-1_Hasenfeld_lane1HG002x01PE20392_2_sequence.txt.gz	HighQuality
HWVTJAFXY_HG002x1xHG002x2_19s005136-1-1_Hasenfeld_lane1HG002x01PE20393_1_sequence.txt.gz	HWVTJAFXY_HG002x1xHG002x2_19s005136-1-1_Hasenfeld_lane1HG002x01PE20393_2_sequence.txt.gz	HighQuality
HWVTJAFXY_HG002x1xHG002x2_19s005136-1-1_Hasenfeld_lane1HG002x02PE20403_1_sequence.txt.gz	HWVTJAFXY_HG002x1xHG002x2_19s005136-1-1_Hasenfeld_lane1HG002x02PE20403_2_sequence.txt.gz	HighQuality
HWVTJAFXY_HG002x1xHG002x2_19s005136-1-1_Hasenfeld_lane1HG002x02PE20405_1_sequence.txt.gz	HWVTJAFXY_HG002x1xHG002x2_19s005136-1-1_Hasenfeld_lane1HG002x02PE20405_2_sequence.txt.gz	HighQuality
HWVTJAFXY_HG002x1xHG002x2_19s005136-1-1_Hasenfeld_lane1HG002x02PE20407_1_sequence.txt.gz	HWVTJAFXY_HG002x1xHG002x2_19s005136-1-1_Hasenfeld_lane1HG002x02PE20407_2_sequence.txt.gz	HighQuality
HWVTJAFXY_HG002x1xHG002x2_19s005136-1-1_Hasenfeld_lane1HG002x02PE20414_1_sequence.txt.gz	HWVTJAFXY_HG002x1xHG002x2_19s005136-1-1_Hasenfeld_lane1HG002x02PE20414_2_sequence.txt.gz	HighQuality
HWVTJAFXY_HG002x1xHG002x2_19s005136-1-1_Hasenfeld_lane1HG002x02PE20416_1_sequence.txt.gz	HWVTJAFXY_HG002x1xHG002x2_19s005136-1-1_Hasenfeld_lane1HG002x02PE20416_2_sequence.txt.gz	HighQuality

HWVTJAFXY_HG002x1xHG002x2_19s005136-1-1_Hasenfeld_lane1HG002x02PE20417_1_sequence.txt.gz	HWVTJAFXY_HG002x1xHG002x2_19s005136-1-1_Hasenfeld_lane1HG002x02PE20417_2_sequence.txt.gz	HighQuality
HWVTJAFXY_HG002x1xHG002x2_19s005136-1-1_Hasenfeld_lane1HG002x02PE20419_1_sequence.txt.gz	HWVTJAFXY_HG002x1xHG002x2_19s005136-1-1_Hasenfeld_lane1HG002x02PE20419_2_sequence.txt.gz	HighQuality
HWVTJAFXY_HG002x1xHG002x2_19s005136-1-1_Hasenfeld_lane1HG002x02PE20421_1_sequence.txt.gz	HWVTJAFXY_HG002x1xHG002x2_19s005136-1-1_Hasenfeld_lane1HG002x02PE20421_2_sequence.txt.gz	HighQuality
HWVTJAFXY_HG002x1xHG002x2_19s005136-1-1_Hasenfeld_lane1HG002x02PE20422_1_sequence.txt.gz	HWVTJAFXY_HG002x1xHG002x2_19s005136-1-1_Hasenfeld_lane1HG002x02PE20422_2_sequence.txt.gz	HighQuality
HWVTJAFXY_HG002x1xHG002x2_19s005136-1-1_Hasenfeld_lane1HG002x02PE20424_1_sequence.txt.gz	HWVTJAFXY_HG002x1xHG002x2_19s005136-1-1_Hasenfeld_lane1HG002x02PE20424_2_sequence.txt.gz	HighQuality
HWVTJAFXY_HG002x1xHG002x2_19s005136-1-1_Hasenfeld_lane1HG002x02PE20425_1_sequence.txt.gz	HWVTJAFXY_HG002x1xHG002x2_19s005136-1-1_Hasenfeld_lane1HG002x02PE20425_2_sequence.txt.gz	HighQuality
HWVTJAFXY_HG002x1xHG002x2_19s005136-1-1_Hasenfeld_lane1HG002x02PE20428_1_sequence.txt.gz	HWVTJAFXY_HG002x1xHG002x2_19s005136-1-1_Hasenfeld_lane1HG002x02PE20428_2_sequence.txt.gz	HighQuality
HWVTJAFXY_HG002x1xHG002x2_19s005136-1-1_Hasenfeld_lane1HG002x02PE20430_1_sequence.txt.gz	HWVTJAFXY_HG002x1xHG002x2_19s005136-1-1_Hasenfeld_lane1HG002x02PE20430_2_sequence.txt.gz	HighQuality
HWVTJAFXY_HG002x1xHG002x2_19s005136-1-1_Hasenfeld_lane1HG002x02PE20431_1_sequence.txt.gz	HWVTJAFXY_HG002x1xHG002x2_19s005136-1-1_Hasenfeld_lane1HG002x02PE20431_2_sequence.txt.gz	HighQuality
HWVTJAFXY_HG002x1xHG002x2_19s005136-1-1_Hasenfeld_lane1HG002x02PE20432_1_sequence.txt.gz	HWVTJAFXY_HG002x1xHG002x2_19s005136-1-1_Hasenfeld_lane1HG002x02PE20432_2_sequence.txt.gz	HighQuality
HWVTJAFXY_HG002x1xHG002x2_19s005136-1-1_Hasenfeld_lane1HG002x02PE20434_1_sequence.txt.gz	HWVTJAFXY_HG002x1xHG002x2_19s005136-1-1_Hasenfeld_lane1HG002x02PE20434_2_sequence.txt.gz	HighQuality
HWVTJAFXY_HG002x1xHG002x2_19s005136-1-1_Hasenfeld_lane1HG002x02PE20435_1_sequence.txt.gz	HWVTJAFXY_HG002x1xHG002x2_19s005136-1-1_Hasenfeld_lane1HG002x02PE20435_2_sequence.txt.gz	HighQuality
HWVTJAFXY_HG002x1xHG002x2_19s005136-1-1_Hasenfeld_lane1HG002x02PE20439_1_sequence.txt.gz	HWVTJAFXY_HG002x1xHG002x2_19s005136-1-1_Hasenfeld_lane1HG002x02PE20439_2_sequence.txt.gz	HighQuality
HWVTJAFXY_HG002x1xHG002x2_19s005136-1-1_Hasenfeld_lane1HG002x02PE20440_1_sequence.txt.gz	HWVTJAFXY_HG002x1xHG002x2_19s005136-1-1_Hasenfeld_lane1HG002x02PE20440_2_sequence.txt.gz	HighQuality
HWVTJAFXY_HG002x1xHG002x2_19s005136-1-1_Hasenfeld_lane1HG002x02PE20443_1_sequence.txt.gz	HWVTJAFXY_HG002x1xHG002x2_19s005136-1-1_Hasenfeld_lane1HG002x02PE20443_2_sequence.txt.gz	HighQuality
HWVTJAFXY_HG002x1xHG002x2_19s005136-1-1_Hasenfeld_lane1HG002x02PE20445_1_sequence.txt.gz	HWVTJAFXY_HG002x1xHG002x2_19s005136-1-1_Hasenfeld_lane1HG002x02PE20445_2_sequence.txt.gz	HighQuality
HWVTJAFXY_HG002x1xHG002x2_19s005136-1-1_Hasenfeld_lane1HG002x02PE20450_1_sequence.txt.gz	HWVTJAFXY_HG002x1xHG002x2_19s005136-1-1_Hasenfeld_lane1HG002x02PE20450_2_sequence.txt.gz	HighQuality
HWVTJAFXY_HG002x1xHG002x2_19s005136-1-1_Hasenfeld_lane1HG002x02PE20452_1_sequence.txt.gz	HWVTJAFXY_HG002x1xHG002x2_19s005136-1-1_Hasenfeld_lane1HG002x02PE20452_2_sequence.txt.gz	HighQuality
HWVTJAFXY_HG002x1xHG002x2_19s005136-1-1_Hasenfeld_lane1HG002x02PE20454_1_sequence.txt.gz	HWVTJAFXY_HG002x1xHG002x2_19s005136-1-1_Hasenfeld_lane1HG002x02PE20454_2_sequence.txt.gz	HighQuality
HWVTJAFXY_HG002x1xHG002x2_19s005136-1-1_Hasenfeld_lane1HG002x02PE20456_1_sequence.txt.gz	HWVTJAFXY_HG002x1xHG002x2_19s005136-1-1_Hasenfeld_lane1HG002x02PE20456_2_sequence.txt.gz	HighQuality
HWVTJAFXY_HG002x1xHG002x2_19s005136-1-1_Hasenfeld_lane1HG002x02PE20458_1_sequence.txt.gz	HWVTJAFXY_HG002x1xHG002x2_19s005136-1-1_Hasenfeld_lane1HG002x02PE20458_2_sequence.txt.gz	HighQuality
HWVTJAFXY_HG002x1xHG002x2_19s005136-1-1_Hasenfeld_lane1HG002x02PE20464_1_sequence.txt.gz	HWVTJAFXY_HG002x1xHG002x2_19s005136-1-1_Hasenfeld_lane1HG002x02PE20464_2_sequence.txt.gz	HighQuality
HWVTJAFXY_HG002x1xHG002x2_19s005136-1-1_Hasenfeld_lane1HG002x02PE20466_1_sequence.txt.gz	HWVTJAFXY_HG002x1xHG002x2_19s005136-1-1_Hasenfeld_lane1HG002x02PE20466_2_sequence.txt.gz	HighQuality
HWVTJAFXY_HG002x1xHG002x2_19s005136-1-1_Hasenfeld_lane1HG002x02PE20467_1_sequence.txt.gz	HWVTJAFXY_HG002x1xHG002x2_19s005136-1-1_Hasenfeld_lane1HG002x02PE20467_2_sequence.txt.gz	HighQuality
HWVTJAFXY_HG002x1xHG002x2_19s005136-1-1_Hasenfeld_lane1HG002x02PE20469_1_sequence.txt.gz	HWVTJAFXY_HG002x1xHG002x2_19s005136-1-1_Hasenfeld_lane1HG002x02PE20469_2_sequence.txt.gz	HighQuality

HWVTJAFXY_HG002x1xHG002x2_19s005136-1-1_Hasenfeld_lane1HG002x02PE20473_1_sequence.txt.gz	HWVTJAFXY_HG002x1xHG002x2_19s005136-1-1_Hasenfeld_lane1HG002x02PE20473_2_sequence.txt.gz	HighQuality
HWVTJAFXY_HG002x1xHG002x2_19s005136-1-1_Hasenfeld_lane1HG002x02PE20479_1_sequence.txt.gz	HWVTJAFXY_HG002x1xHG002x2_19s005136-1-1_Hasenfeld_lane1HG002x02PE20479_2_sequence.txt.gz	HighQuality
HWVTJAFXY_HG002x1xHG002x2_19s005136-1-1_Hasenfeld_lane1HG002x02PE20483_1_sequence.txt.gz	HWVTJAFXY_HG002x1xHG002x2_19s005136-1-1_Hasenfeld_lane1HG002x02PE20483_2_sequence.txt.gz	HighQuality
HWVTJAFXY_HG002x1xHG002x2_19s005136-1-1_Hasenfeld_lane1HG002x02PE20484_1_sequence.txt.gz	HWVTJAFXY_HG002x1xHG002x2_19s005136-1-1_Hasenfeld_lane1HG002x02PE20484_2_sequence.txt.gz	HighQuality
HWVTJAFXY_HG002x1xHG002x2_19s005136-1-1_Hasenfeld_lane1HG002x02PE20485_1_sequence.txt.gz	HWVTJAFXY_HG002x1xHG002x2_19s005136-1-1_Hasenfeld_lane1HG002x02PE20485_2_sequence.txt.gz	HighQuality
HWVTJAFXY_HG002x1xHG002x2_19s005136-1-1_Hasenfeld_lane1HG002x02PE20486_1_sequence.txt.gz	HWVTJAFXY_HG002x1xHG002x2_19s005136-1-1_Hasenfeld_lane1HG002x02PE20486_2_sequence.txt.gz	HighQuality
HWVTJAFXY_HG002x1xHG002x2_19s005136-1-1_Hasenfeld_lane1HG002x02PE20487_1_sequence.txt.gz	HWVTJAFXY_HG002x1xHG002x2_19s005136-1-1_Hasenfeld_lane1HG002x02PE20487_2_sequence.txt.gz	HighQuality
HWVTJAFXY_HG002x1xHG002x2_19s005136-1-1_Hasenfeld_lane1HG002x02PE20488_1_sequence.txt.gz	HWVTJAFXY_HG002x1xHG002x2_19s005136-1-1_Hasenfeld_lane1HG002x02PE20488_2_sequence.txt.gz	HighQuality
HWVTJAFXY_HG002x1xHG002x2_19s005136-1-1_Hasenfeld_lane1HG002x02PE20490_1_sequence.txt.gz	HWVTJAFXY_HG002x1xHG002x2_19s005136-1-1_Hasenfeld_lane1HG002x02PE20490_2_sequence.txt.gz	HighQuality
HWVTJAFXY_HG002x1xHG002x2_19s005136-1-1_Hasenfeld_lane1HG002x02PE20491_1_sequence.txt.gz	HWVTJAFXY_HG002x1xHG002x2_19s005136-1-1_Hasenfeld_lane1HG002x02PE20491_2_sequence.txt.gz	HighQuality
HWVTJAFXY_HG002x1xHG002x2_19s005136-1-1_Hasenfeld_lane1HG002x02PE20492_1_sequence.txt.gz	HWVTJAFXY_HG002x1xHG002x2_19s005136-1-1_Hasenfeld_lane1HG002x02PE20492_2_sequence.txt.gz	HighQuality
HWVTJAFXY_HG002x1xHG002x2_19s005136-1-1_Hasenfeld_lane1HG002x02PE20494_1_sequence.txt.gz	HWVTJAFXY_HG002x1xHG002x2_19s005136-1-1_Hasenfeld_lane1HG002x02PE20494_2_sequence.txt.gz	HighQuality
HWVTJAFXY_HG002x1xHG002x2_19s005136-1-1_Hasenfeld_lane1HG002x01PE20302_1_sequence.txt.gz	HWVTJAFXY_HG002x1xHG002x2_19s005136-1-1_Hasenfeld_lane1HG002x01PE20302_2_sequence.txt.gz	NotHighQuality
HWVTJAFXY_HG002x1xHG002x2_19s005136-1-1_Hasenfeld_lane1HG002x01PE20304_1_sequence.txt.gz	HWVTJAFXY_HG002x1xHG002x2_19s005136-1-1_Hasenfeld_lane1HG002x01PE20304_2_sequence.txt.gz	NotHighQuality
HWVTJAFXY_HG002x1xHG002x2_19s005136-1-1_Hasenfeld_lane1HG002x01PE20309_1_sequence.txt.gz	HWVTJAFXY_HG002x1xHG002x2_19s005136-1-1_Hasenfeld_lane1HG002x01PE20309_2_sequence.txt.gz	NotHighQuality
HWVTJAFXY_HG002x1xHG002x2_19s005136-1-1_Hasenfeld_lane1HG002x01PE20310_1_sequence.txt.gz	HWVTJAFXY_HG002x1xHG002x2_19s005136-1-1_Hasenfeld_lane1HG002x01PE20310_2_sequence.txt.gz	NotHighQuality
HWVTJAFXY_HG002x1xHG002x2_19s005136-1-1_Hasenfeld_lane1HG002x01PE20311_1_sequence.txt.gz	HWVTJAFXY_HG002x1xHG002x2_19s005136-1-1_Hasenfeld_lane1HG002x01PE20311_2_sequence.txt.gz	NotHighQuality
HWVTJAFXY_HG002x1xHG002x2_19s005136-1-1_Hasenfeld_lane1HG002x01PE20312_1_sequence.txt.gz	HWVTJAFXY_HG002x1xHG002x2_19s005136-1-1_Hasenfeld_lane1HG002x01PE20312_2_sequence.txt.gz	NotHighQuality
HWVTJAFXY_HG002x1xHG002x2_19s005136-1-1_Hasenfeld_lane1HG002x01PE20314_1_sequence.txt.gz	HWVTJAFXY_HG002x1xHG002x2_19s005136-1-1_Hasenfeld_lane1HG002x01PE20314_2_sequence.txt.gz	NotHighQuality
HWVTJAFXY_HG002x1xHG002x2_19s005136-1-1_Hasenfeld_lane1HG002x01PE20316_1_sequence.txt.gz	HWVTJAFXY_HG002x1xHG002x2_19s005136-1-1_Hasenfeld_lane1HG002x01PE20316_2_sequence.txt.gz	NotHighQuality
HWVTJAFXY_HG002x1xHG002x2_19s005136-1-1_Hasenfeld_lane1HG002x01PE20317_1_sequence.txt.gz	HWVTJAFXY_HG002x1xHG002x2_19s005136-1-1_Hasenfeld_lane1HG002x01PE20317_2_sequence.txt.gz	NotHighQuality
HWVTJAFXY_HG002x1xHG002x2_19s005136-1-1_Hasenfeld_lane1HG002x01PE20320_1_sequence.txt.gz	HWVTJAFXY_HG002x1xHG002x2_19s005136-1-1_Hasenfeld_lane1HG002x01PE20320_2_sequence.txt.gz	NotHighQuality
HWVTJAFXY_HG002x1xHG002x2_19s005136-1-1_Hasenfeld_lane1HG002x01PE20321_1_sequence.txt.gz	HWVTJAFXY_HG002x1xHG002x2_19s005136-1-1_Hasenfeld_lane1HG002x01PE20321_2_sequence.txt.gz	NotHighQuality
HWVTJAFXY_HG002x1xHG002x2_19s005136-1-1_Hasenfeld_lane1HG002x01PE20322_1_sequence.txt.gz	HWVTJAFXY_HG002x1xHG002x2_19s005136-1-1_Hasenfeld_lane1HG002x01PE20322_2_sequence.txt.gz	NotHighQuality
HWVTJAFXY_HG002x1xHG002x2_19s005136-1-1_Hasenfeld_lane1HG002x01PE20323_1_sequence.txt.gz	HWVTJAFXY_HG002x1xHG002x2_19s005136-1-1_Hasenfeld_lane1HG002x01PE20323_2_sequence.txt.gz	NotHighQuality

HWVTJAFXY_HG002x1xHG002x2_19s005136-1-1_Hasenfeld_lane1HG002x01PE20324_1_sequence.txt.gz	HWVTJAFXY_HG002x1xHG002x2_19s005136-1-1_Hasenfeld_lane1HG002x01PE20324_2_sequence.txt.gz	NotHighQuality
HWVTJAFXY_HG002x1xHG002x2_19s005136-1-1_Hasenfeld_lane1HG002x01PE20326_1_sequence.txt.gz	HWVTJAFXY_HG002x1xHG002x2_19s005136-1-1_Hasenfeld_lane1HG002x01PE20326_2_sequence.txt.gz	NotHighQuality
HWVTJAFXY_HG002x1xHG002x2_19s005136-1-1_Hasenfeld_lane1HG002x01PE20330_1_sequence.txt.gz	HWVTJAFXY_HG002x1xHG002x2_19s005136-1-1_Hasenfeld_lane1HG002x01PE20330_2_sequence.txt.gz	NotHighQuality
HWVTJAFXY_HG002x1xHG002x2_19s005136-1-1_Hasenfeld_lane1HG002x01PE20333_1_sequence.txt.gz	HWVTJAFXY_HG002x1xHG002x2_19s005136-1-1_Hasenfeld_lane1HG002x01PE20333_2_sequence.txt.gz	NotHighQuality
HWVTJAFXY_HG002x1xHG002x2_19s005136-1-1_Hasenfeld_lane1HG002x01PE20338_1_sequence.txt.gz	HWVTJAFXY_HG002x1xHG002x2_19s005136-1-1_Hasenfeld_lane1HG002x01PE20338_2_sequence.txt.gz	NotHighQuality
HWVTJAFXY_HG002x1xHG002x2_19s005136-1-1_Hasenfeld_lane1HG002x01PE20344_1_sequence.txt.gz	HWVTJAFXY_HG002x1xHG002x2_19s005136-1-1_Hasenfeld_lane1HG002x01PE20344_2_sequence.txt.gz	NotHighQuality
HWVTJAFXY_HG002x1xHG002x2_19s005136-1-1_Hasenfeld_lane1HG002x01PE20346_1_sequence.txt.gz	HWVTJAFXY_HG002x1xHG002x2_19s005136-1-1_Hasenfeld_lane1HG002x01PE20346_2_sequence.txt.gz	NotHighQuality
HWVTJAFXY_HG002x1xHG002x2_19s005136-1-1_Hasenfeld_lane1HG002x01PE20348_1_sequence.txt.gz	HWVTJAFXY_HG002x1xHG002x2_19s005136-1-1_Hasenfeld_lane1HG002x01PE20348_2_sequence.txt.gz	NotHighQuality
HWVTJAFXY_HG002x1xHG002x2_19s005136-1-1_Hasenfeld_lane1HG002x01PE20349_1_sequence.txt.gz	HWVTJAFXY_HG002x1xHG002x2_19s005136-1-1_Hasenfeld_lane1HG002x01PE20349_2_sequence.txt.gz	NotHighQuality
HWVTJAFXY_HG002x1xHG002x2_19s005136-1-1_Hasenfeld_lane1HG002x01PE20354_1_sequence.txt.gz	HWVTJAFXY_HG002x1xHG002x2_19s005136-1-1_Hasenfeld_lane1HG002x01PE20354_2_sequence.txt.gz	NotHighQuality
HWVTJAFXY_HG002x1xHG002x2_19s005136-1-1_Hasenfeld_lane1HG002x01PE20360_1_sequence.txt.gz	HWVTJAFXY_HG002x1xHG002x2_19s005136-1-1_Hasenfeld_lane1HG002x01PE20360_2_sequence.txt.gz	NotHighQuality
HWVTJAFXY_HG002x1xHG002x2_19s005136-1-1_Hasenfeld_lane1HG002x01PE20365_1_sequence.txt.gz	HWVTJAFXY_HG002x1xHG002x2_19s005136-1-1_Hasenfeld_lane1HG002x01PE20365_2_sequence.txt.gz	NotHighQuality
HWVTJAFXY_HG002x1xHG002x2_19s005136-1-1_Hasenfeld_lane1HG002x01PE20366_1_sequence.txt.gz	HWVTJAFXY_HG002x1xHG002x2_19s005136-1-1_Hasenfeld_lane1HG002x01PE20366_2_sequence.txt.gz	NotHighQuality
HWVTJAFXY_HG002x1xHG002x2_19s005136-1-1_Hasenfeld_lane1HG002x01PE20369_1_sequence.txt.gz	HWVTJAFXY_HG002x1xHG002x2_19s005136-1-1_Hasenfeld_lane1HG002x01PE20369_2_sequence.txt.gz	NotHighQuality
HWVTJAFXY_HG002x1xHG002x2_19s005136-1-1_Hasenfeld_lane1HG002x01PE20370_1_sequence.txt.gz	HWVTJAFXY_HG002x1xHG002x2_19s005136-1-1_Hasenfeld_lane1HG002x01PE20370_2_sequence.txt.gz	NotHighQuality
HWVTJAFXY_HG002x1xHG002x2_19s005136-1-1_Hasenfeld_lane1HG002x01PE20371_1_sequence.txt.gz	HWVTJAFXY_HG002x1xHG002x2_19s005136-1-1_Hasenfeld_lane1HG002x01PE20371_2_sequence.txt.gz	NotHighQuality
HWVTJAFXY_HG002x1xHG002x2_19s005136-1-1_Hasenfeld_lane1HG002x01PE20372_1_sequence.txt.gz	HWVTJAFXY_HG002x1xHG002x2_19s005136-1-1_Hasenfeld_lane1HG002x01PE20372_2_sequence.txt.gz	NotHighQuality
HWVTJAFXY_HG002x1xHG002x2_19s005136-1-1_Hasenfeld_lane1HG002x01PE20373_1_sequence.txt.gz	HWVTJAFXY_HG002x1xHG002x2_19s005136-1-1_Hasenfeld_lane1HG002x01PE20373_2_sequence.txt.gz	NotHighQuality
HWVTJAFXY_HG002x1xHG002x2_19s005136-1-1_Hasenfeld_lane1HG002x01PE20375_1_sequence.txt.gz	HWVTJAFXY_HG002x1xHG002x2_19s005136-1-1_Hasenfeld_lane1HG002x01PE20375_2_sequence.txt.gz	NotHighQuality
HWVTJAFXY_HG002x1xHG002x2_19s005136-1-1_Hasenfeld_lane1HG002x01PE20380_1_sequence.txt.gz	HWVTJAFXY_HG002x1xHG002x2_19s005136-1-1_Hasenfeld_lane1HG002x01PE20380_2_sequence.txt.gz	NotHighQuality
HWVTJAFXY_HG002x1xHG002x2_19s005136-1-1_Hasenfeld_lane1HG002x01PE20382_1_sequence.txt.gz	HWVTJAFXY_HG002x1xHG002x2_19s005136-1-1_Hasenfeld_lane1HG002x01PE20382_2_sequence.txt.gz	NotHighQuality
HWVTJAFXY_HG002x1xHG002x2_19s005136-1-1_Hasenfeld_lane1HG002x01PE20383_1_sequence.txt.gz	HWVTJAFXY_HG002x1xHG002x2_19s005136-1-1_Hasenfeld_lane1HG002x01PE20383_2_sequence.txt.gz	NotHighQuality
HWVTJAFXY_HG002x1xHG002x2_19s005136-1-1_Hasenfeld_lane1HG002x01PE20384_1_sequence.txt.gz	HWVTJAFXY_HG002x1xHG002x2_19s005136-1-1_Hasenfeld_lane1HG002x01PE20384_2_sequence.txt.gz	NotHighQuality
HWVTJAFXY_HG002x1xHG002x2_19s005136-1-1_Hasenfeld_lane1HG002x01PE20385_1_sequence.txt.gz	HWVTJAFXY_HG002x1xHG002x2_19s005136-1-1_Hasenfeld_lane1HG002x01PE20385_2_sequence.txt.gz	NotHighQuality
HWVTJAFXY_HG002x1xHG002x2_19s005136-1-1_Hasenfeld_lane1HG002x01PE20386_1_sequence.txt.gz	HWVTJAFXY_HG002x1xHG002x2_19s005136-1-1_Hasenfeld_lane1HG002x01PE20386_2_sequence.txt.gz	NotHighQuality

HWVTJAFXY_HG002x1xHG002x2_19s005136-1-1_Hasenfeld_lane1HG002x01PE20390_1_sequence.txt.gz	HWVTJAFXY_HG002x1xHG002x2_19s005136-1-1_Hasenfeld_lane1HG002x01PE20390_2_sequence.txt.gz	NotHighQuality
HWVTJAFXY_HG002x1xHG002x2_19s005136-1-1_Hasenfeld_lane1HG002x01PE20394_1_sequence.txt.gz	HWVTJAFXY_HG002x1xHG002x2_19s005136-1-1_Hasenfeld_lane1HG002x01PE20394_2_sequence.txt.gz	NotHighQuality
HWVTJAFXY_HG002x1xHG002x2_19s005136-1-1_Hasenfeld_lane1HG002x01PE20395_1_sequence.txt.gz	HWVTJAFXY_HG002x1xHG002x2_19s005136-1-1_Hasenfeld_lane1HG002x01PE20395_2_sequence.txt.gz	NotHighQuality
HWVTJAFXY_HG002x1xHG002x2_19s005136-1-1_Hasenfeld_lane1HG002x01PE20396_1_sequence.txt.gz	HWVTJAFXY_HG002x1xHG002x2_19s005136-1-1_Hasenfeld_lane1HG002x01PE20396_2_sequence.txt.gz	NotHighQuality
HWVTJAFXY_HG002x1xHG002x2_19s005136-1-1_Hasenfeld_lane1HG002x02PE20401_1_sequence.txt.gz	HWVTJAFXY_HG002x1xHG002x2_19s005136-1-1_Hasenfeld_lane1HG002x02PE20401_2_sequence.txt.gz	NotHighQuality
HWVTJAFXY_HG002x1xHG002x2_19s005136-1-1_Hasenfeld_lane1HG002x02PE20402_1_sequence.txt.gz	HWVTJAFXY_HG002x1xHG002x2_19s005136-1-1_Hasenfeld_lane1HG002x02PE20402_2_sequence.txt.gz	NotHighQuality
HWVTJAFXY_HG002x1xHG002x2_19s005136-1-1_Hasenfeld_lane1HG002x02PE20404_1_sequence.txt.gz	HWVTJAFXY_HG002x1xHG002x2_19s005136-1-1_Hasenfeld_lane1HG002x02PE20404_2_sequence.txt.gz	NotHighQuality
HWVTJAFXY_HG002x1xHG002x2_19s005136-1-1_Hasenfeld_lane1HG002x02PE20406_1_sequence.txt.gz	HWVTJAFXY_HG002x1xHG002x2_19s005136-1-1_Hasenfeld_lane1HG002x02PE20406_2_sequence.txt.gz	NotHighQuality
HWVTJAFXY_HG002x1xHG002x2_19s005136-1-1_Hasenfeld_lane1HG002x02PE20408_1_sequence.txt.gz	HWVTJAFXY_HG002x1xHG002x2_19s005136-1-1_Hasenfeld_lane1HG002x02PE20408_2_sequence.txt.gz	NotHighQuality
HWVTJAFXY_HG002x1xHG002x2_19s005136-1-1_Hasenfeld_lane1HG002x02PE20409_1_sequence.txt.gz	HWVTJAFXY_HG002x1xHG002x2_19s005136-1-1_Hasenfeld_lane1HG002x02PE20409_2_sequence.txt.gz	NotHighQuality
HWVTJAFXY_HG002x1xHG002x2_19s005136-1-1_Hasenfeld_lane1HG002x02PE20410_1_sequence.txt.gz	HWVTJAFXY_HG002x1xHG002x2_19s005136-1-1_Hasenfeld_lane1HG002x02PE20410_2_sequence.txt.gz	NotHighQuality
HWVTJAFXY_HG002x1xHG002x2_19s005136-1-1_Hasenfeld_lane1HG002x02PE20411_1_sequence.txt.gz	HWVTJAFXY_HG002x1xHG002x2_19s005136-1-1_Hasenfeld_lane1HG002x02PE20411_2_sequence.txt.gz	NotHighQuality
HWVTJAFXY_HG002x1xHG002x2_19s005136-1-1_Hasenfeld_lane1HG002x02PE20412_1_sequence.txt.gz	HWVTJAFXY_HG002x1xHG002x2_19s005136-1-1_Hasenfeld_lane1HG002x02PE20412_2_sequence.txt.gz	NotHighQuality
HWVTJAFXY_HG002x1xHG002x2_19s005136-1-1_Hasenfeld_lane1HG002x02PE20413_1_sequence.txt.gz	HWVTJAFXY_HG002x1xHG002x2_19s005136-1-1_Hasenfeld_lane1HG002x02PE20413_2_sequence.txt.gz	NotHighQuality
HWVTJAFXY_HG002x1xHG002x2_19s005136-1-1_Hasenfeld_lane1HG002x02PE20415_1_sequence.txt.gz	HWVTJAFXY_HG002x1xHG002x2_19s005136-1-1_Hasenfeld_lane1HG002x02PE20415_2_sequence.txt.gz	NotHighQuality
HWVTJAFXY_HG002x1xHG002x2_19s005136-1-1_Hasenfeld_lane1HG002x02PE20418_1_sequence.txt.gz	HWVTJAFXY_HG002x1xHG002x2_19s005136-1-1_Hasenfeld_lane1HG002x02PE20418_2_sequence.txt.gz	NotHighQuality
HWVTJAFXY_HG002x1xHG002x2_19s005136-1-1_Hasenfeld_lane1HG002x02PE20420_1_sequence.txt.gz	HWVTJAFXY_HG002x1xHG002x2_19s005136-1-1_Hasenfeld_lane1HG002x02PE20420_2_sequence.txt.gz	NotHighQuality
HWVTJAFXY_HG002x1xHG002x2_19s005136-1-1_Hasenfeld_lane1HG002x02PE20423_1_sequence.txt.gz	HWVTJAFXY_HG002x1xHG002x2_19s005136-1-1_Hasenfeld_lane1HG002x02PE20423_2_sequence.txt.gz	NotHighQuality
HWVTJAFXY_HG002x1xHG002x2_19s005136-1-1_Hasenfeld_lane1HG002x02PE20426_1_sequence.txt.gz	HWVTJAFXY_HG002x1xHG002x2_19s005136-1-1_Hasenfeld_lane1HG002x02PE20426_2_sequence.txt.gz	NotHighQuality
HWVTJAFXY_HG002x1xHG002x2_19s005136-1-1_Hasenfeld_lane1HG002x02PE20427_1_sequence.txt.gz	HWVTJAFXY_HG002x1xHG002x2_19s005136-1-1_Hasenfeld_lane1HG002x02PE20427_2_sequence.txt.gz	NotHighQuality
HWVTJAFXY_HG002x1xHG002x2_19s005136-1-1_Hasenfeld_lane1HG002x02PE20429_1_sequence.txt.gz	HWVTJAFXY_HG002x1xHG002x2_19s005136-1-1_Hasenfeld_lane1HG002x02PE20429_2_sequence.txt.gz	NotHighQuality
HWVTJAFXY_HG002x1xHG002x2_19s005136-1-1_Hasenfeld_lane1HG002x02PE20433_1_sequence.txt.gz	HWVTJAFXY_HG002x1xHG002x2_19s005136-1-1_Hasenfeld_lane1HG002x02PE20433_2_sequence.txt.gz	NotHighQuality
HWVTJAFXY_HG002x1xHG002x2_19s005136-1-1_Hasenfeld_lane1HG002x02PE20436_1_sequence.txt.gz	HWVTJAFXY_HG002x1xHG002x2_19s005136-1-1_Hasenfeld_lane1HG002x02PE20436_2_sequence.txt.gz	NotHighQuality
HWVTJAFXY_HG002x1xHG002x2_19s005136-1-1_Hasenfeld_lane1HG002x02PE20437_1_sequence.txt.gz	HWVTJAFXY_HG002x1xHG002x2_19s005136-1-1_Hasenfeld_lane1HG002x02PE20437_2_sequence.txt.gz	NotHighQuality
HWVTJAFXY_HG002x1xHG002x2_19s005136-1-1_Hasenfeld_lane1HG002x02PE20438_1_sequence.txt.gz	HWVTJAFXY_HG002x1xHG002x2_19s005136-1-1_Hasenfeld_lane1HG002x02PE20438_2_sequence.txt.gz	NotHighQuality

HWVTJAFXY_HG002x1xHG002x2_19s005136-1-1_Hasenfeld_lane1HG002x02PE20441_1_sequence.txt.gz	HWVTJAFXY_HG002x1xHG002x2_19s005136-1-1_Hasenfeld_lane1HG002x02PE20441_2_sequence.txt.gz	NotHighQuality
HWVTJAFXY_HG002x1xHG002x2_19s005136-1-1_Hasenfeld_lane1HG002x02PE20442_1_sequence.txt.gz	HWVTJAFXY_HG002x1xHG002x2_19s005136-1-1_Hasenfeld_lane1HG002x02PE20442_2_sequence.txt.gz	NotHighQuality
HWVTJAFXY_HG002x1xHG002x2_19s005136-1-1_Hasenfeld_lane1HG002x02PE20444_1_sequence.txt.gz	HWVTJAFXY_HG002x1xHG002x2_19s005136-1-1_Hasenfeld_lane1HG002x02PE20444_2_sequence.txt.gz	NotHighQuality
HWVTJAFXY_HG002x1xHG002x2_19s005136-1-1_Hasenfeld_lane1HG002x02PE20446_1_sequence.txt.gz	HWVTJAFXY_HG002x1xHG002x2_19s005136-1-1_Hasenfeld_lane1HG002x02PE20446_2_sequence.txt.gz	NotHighQuality
HWVTJAFXY_HG002x1xHG002x2_19s005136-1-1_Hasenfeld_lane1HG002x02PE20447_1_sequence.txt.gz	HWVTJAFXY_HG002x1xHG002x2_19s005136-1-1_Hasenfeld_lane1HG002x02PE20447_2_sequence.txt.gz	NotHighQuality
HWVTJAFXY_HG002x1xHG002x2_19s005136-1-1_Hasenfeld_lane1HG002x02PE20448_1_sequence.txt.gz	HWVTJAFXY_HG002x1xHG002x2_19s005136-1-1_Hasenfeld_lane1HG002x02PE20448_2_sequence.txt.gz	NotHighQuality
HWVTJAFXY_HG002x1xHG002x2_19s005136-1-1_Hasenfeld_lane1HG002x02PE20449_1_sequence.txt.gz	HWVTJAFXY_HG002x1xHG002x2_19s005136-1-1_Hasenfeld_lane1HG002x02PE20449_2_sequence.txt.gz	NotHighQuality
HWVTJAFXY_HG002x1xHG002x2_19s005136-1-1_Hasenfeld_lane1HG002x02PE20451_1_sequence.txt.gz	HWVTJAFXY_HG002x1xHG002x2_19s005136-1-1_Hasenfeld_lane1HG002x02PE20451_2_sequence.txt.gz	NotHighQuality
HWVTJAFXY_HG002x1xHG002x2_19s005136-1-1_Hasenfeld_lane1HG002x02PE20453_1_sequence.txt.gz	HWVTJAFXY_HG002x1xHG002x2_19s005136-1-1_Hasenfeld_lane1HG002x02PE20453_2_sequence.txt.gz	NotHighQuality
HWVTJAFXY_HG002x1xHG002x2_19s005136-1-1_Hasenfeld_lane1HG002x02PE20455_1_sequence.txt.gz	HWVTJAFXY_HG002x1xHG002x2_19s005136-1-1_Hasenfeld_lane1HG002x02PE20455_2_sequence.txt.gz	NotHighQuality
HWVTJAFXY_HG002x1xHG002x2_19s005136-1-1_Hasenfeld_lane1HG002x02PE20457_1_sequence.txt.gz	HWVTJAFXY_HG002x1xHG002x2_19s005136-1-1_Hasenfeld_lane1HG002x02PE20457_2_sequence.txt.gz	NotHighQuality
HWVTJAFXY_HG002x1xHG002x2_19s005136-1-1_Hasenfeld_lane1HG002x02PE20459_1_sequence.txt.gz	HWVTJAFXY_HG002x1xHG002x2_19s005136-1-1_Hasenfeld_lane1HG002x02PE20459_2_sequence.txt.gz	NotHighQuality
HWVTJAFXY_HG002x1xHG002x2_19s005136-1-1_Hasenfeld_lane1HG002x02PE20460_1_sequence.txt.gz	HWVTJAFXY_HG002x1xHG002x2_19s005136-1-1_Hasenfeld_lane1HG002x02PE20460_2_sequence.txt.gz	NotHighQuality
HWVTJAFXY_HG002x1xHG002x2_19s005136-1-1_Hasenfeld_lane1HG002x02PE20461_1_sequence.txt.gz	HWVTJAFXY_HG002x1xHG002x2_19s005136-1-1_Hasenfeld_lane1HG002x02PE20461_2_sequence.txt.gz	NotHighQuality
HWVTJAFXY_HG002x1xHG002x2_19s005136-1-1_Hasenfeld_lane1HG002x02PE20462_1_sequence.txt.gz	HWVTJAFXY_HG002x1xHG002x2_19s005136-1-1_Hasenfeld_lane1HG002x02PE20462_2_sequence.txt.gz	NotHighQuality
HWVTJAFXY_HG002x1xHG002x2_19s005136-1-1_Hasenfeld_lane1HG002x02PE20463_1_sequence.txt.gz	HWVTJAFXY_HG002x1xHG002x2_19s005136-1-1_Hasenfeld_lane1HG002x02PE20463_2_sequence.txt.gz	NotHighQuality
HWVTJAFXY_HG002x1xHG002x2_19s005136-1-1_Hasenfeld_lane1HG002x02PE20465_1_sequence.txt.gz	HWVTJAFXY_HG002x1xHG002x2_19s005136-1-1_Hasenfeld_lane1HG002x02PE20465_2_sequence.txt.gz	NotHighQuality
HWVTJAFXY_HG002x1xHG002x2_19s005136-1-1_Hasenfeld_lane1HG002x02PE20468_1_sequence.txt.gz	HWVTJAFXY_HG002x1xHG002x2_19s005136-1-1_Hasenfeld_lane1HG002x02PE20468_2_sequence.txt.gz	NotHighQuality
HWVTJAFXY_HG002x1xHG002x2_19s005136-1-1_Hasenfeld_lane1HG002x02PE20470_1_sequence.txt.gz	HWVTJAFXY_HG002x1xHG002x2_19s005136-1-1_Hasenfeld_lane1HG002x02PE20470_2_sequence.txt.gz	NotHighQuality
HWVTJAFXY_HG002x1xHG002x2_19s005136-1-1_Hasenfeld_lane1HG002x02PE20471_1_sequence.txt.gz	HWVTJAFXY_HG002x1xHG002x2_19s005136-1-1_Hasenfeld_lane1HG002x02PE20471_2_sequence.txt.gz	NotHighQuality
HWVTJAFXY_HG002x1xHG002x2_19s005136-1-1_Hasenfeld_lane1HG002x02PE20472_1_sequence.txt.gz	HWVTJAFXY_HG002x1xHG002x2_19s005136-1-1_Hasenfeld_lane1HG002x02PE20472_2_sequence.txt.gz	NotHighQuality
HWVTJAFXY_HG002x1xHG002x2_19s005136-1-1_Hasenfeld_lane1HG002x02PE20474_1_sequence.txt.gz	HWVTJAFXY_HG002x1xHG002x2_19s005136-1-1_Hasenfeld_lane1HG002x02PE20474_2_sequence.txt.gz	NotHighQuality
HWVTJAFXY_HG002x1xHG002x2_19s005136-1-1_Hasenfeld_lane1HG002x02PE20475_1_sequence.txt.gz	HWVTJAFXY_HG002x1xHG002x2_19s005136-1-1_Hasenfeld_lane1HG002x02PE20475_2_sequence.txt.gz	NotHighQuality
HWVTJAFXY_HG002x1xHG002x2_19s005136-1-1_Hasenfeld_lane1HG002x02PE20476_1_sequence.txt.gz	HWVTJAFXY_HG002x1xHG002x2_19s005136-1-1_Hasenfeld_lane1HG002x02PE20476_2_sequence.txt.gz	NotHighQuality
HWVTJAFXY_HG002x1xHG002x2_19s005136-1-1_Hasenfeld_lane1HG002x02PE20477_1_sequence.txt.gz	HWVTJAFXY_HG002x1xHG002x2_19s005136-1-1_Hasenfeld_lane1HG002x02PE20477_2_sequence.txt.gz	NotHighQuality

HWVTJAFXY_HG002x1xHG002x2_19s005136-1-1_Hasenfeld_lane1HG002x02PE20478_1_sequence.txt.gz	HWVTJAFXY_HG002x1xHG002x2_19s005136-1-1_Hasenfeld_lane1HG002x02PE20478_2_sequence.txt.gz	NotHighQuality
HWVTJAFXY_HG002x1xHG002x2_19s005136-1-1_Hasenfeld_lane1HG002x02PE20480_1_sequence.txt.gz	HWVTJAFXY_HG002x1xHG002x2_19s005136-1-1_Hasenfeld_lane1HG002x02PE20480_2_sequence.txt.gz	NotHighQuality
HWVTJAFXY_HG002x1xHG002x2_19s005136-1-1_Hasenfeld_lane1HG002x02PE20481_1_sequence.txt.gz	HWVTJAFXY_HG002x1xHG002x2_19s005136-1-1_Hasenfeld_lane1HG002x02PE20481_2_sequence.txt.gz	NotHighQuality
HWVTJAFXY_HG002x1xHG002x2_19s005136-1-1_Hasenfeld_lane1HG002x02PE20482_1_sequence.txt.gz	HWVTJAFXY_HG002x1xHG002x2_19s005136-1-1_Hasenfeld_lane1HG002x02PE20482_2_sequence.txt.gz	NotHighQuality
HWVTJAFXY_HG002x1xHG002x2_19s005136-1-1_Hasenfeld_lane1HG002x02PE20489_1_sequence.txt.gz	HWVTJAFXY_HG002x1xHG002x2_19s005136-1-1_Hasenfeld_lane1HG002x02PE20489_2_sequence.txt.gz	NotHighQuality
HWVTJAFXY_HG002x1xHG002x2_19s005136-1-1_Hasenfeld_lane1HG002x02PE20493_1_sequence.txt.gz	HWVTJAFXY_HG002x1xHG002x2_19s005136-1-1_Hasenfeld_lane1HG002x02PE20493_2_sequence.txt.gz	NotHighQuality
HWVTJAFXY_HG002x1xHG002x2_19s005136-1-1_Hasenfeld_lane1HG002x02PE20495_1_sequence.txt.gz	HWVTJAFXY_HG002x1xHG002x2_19s005136-1-1_Hasenfeld_lane1HG002x02PE20495_2_sequence.txt.gz	NotHighQuality
HWVTJAFXY_HG002x1xHG002x2_19s005136-1-1_Hasenfeld_lane1HG002x02PE20496_1_sequence.txt.gz	HWVTJAFXY_HG002x1xHG002x2_19s005136-1-1_Hasenfeld_lane1HG002x02PE20496_2_sequence.txt.gz	NotHighQuality

Table S2: Verkko NA24385 Titration Scaffold auN:

% High-Quality Libraries	Repetition	Scaffold auN (Mbp hpc)	Fraction of 192 Library Reference auN (%)
0	1	94.7	93.1
25	1	96.9	95.3
25	2	98.1	96.5
25	3	94.0	92.5
25	4	96.5	94.9
50	1	98.0	96.4
50	2	99.2	97.5
50	3	99.9	98.2
50	4	99.0	97.4
75	1	100.0	98.3
75	2	100.0	98.3
75	3	99.2	97.5
75	4	99.8	98.2
100	1	99.0	97.4
